# Benchmarking Swaths of Intermolecular Interaction Components with Symmetry-Adapted Perturbation Theory

**DOI:** 10.1021/acs.jctc.3c00801

**Published:** 2023-12-20

**Authors:** Ehsan Masumian, A. Daniel Boese

**Affiliations:** Physical and Theoretical Chemistry, Department of Chemistry, University of Graz, 8010 Graz, Austria

## Abstract

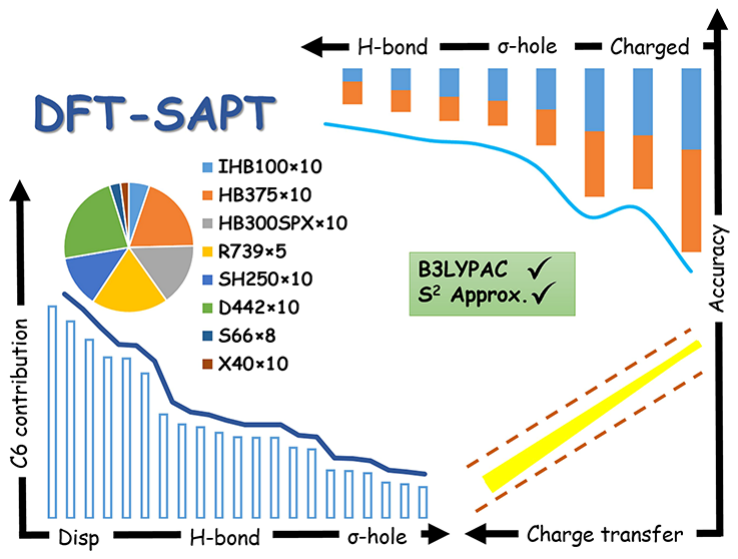

A benchmark database for interaction energy components of various noncovalent interactions (NCIs) along their dissociation curve is one of the essential needs in theoretical chemistry, especially for the development of force fields and machine-learning methods. We utilize DFT-SAPT or SAPT(DFT) as one of the most accurate methods to generate an extensive stock of the energy components, including dispersion energies extrapolated to the complete basis set limit (CBS). Precise analyses of the created data, and benchmarking the total interaction energies against the best available CCSD(T)/CBS values, reveal different aspects of the methodology and the nature of NCIs. For example, error cancellation effects between the *S*^2^ approximation and nonexact xc-potentials occur, and large charge transfer energies in some systems, including heavy atoms, can explain the lower accuracy of DFT-SAPT. This method is perfect for neutral complexes containing light nonmetals, while other systems with heavier atoms should be treated carefully. In the last part, a representative data set for all NCIs is extracted from the original data.

## 1 Introduction

The available benchmark databases with accurate interaction energies are a hotbed of extensive activities for computational and quantum chemists. Standard collections of data help researchers assess approximate methods such as density functional theory (DFT) functionals, find unbiased, reliable data to train and test machine learning models, and probe the nature of chemical phenomena such as noncovalent interactions (NCIs) within a wide range of compounds.

NCIs determine the properties of many chemical systems, including molecular crystals,^1^ solvents,^2^ polymers,^3^ proteins,^4^ and nucleotides.^5^ These interactions also control the self-assembly of nanomaterials,^6^ drug docking processes,^7^ and the binding of reaction partners in supramolecular catalysis.^8^ NCIs are weak contacts compared to covalent bonds and thus very sensitive to the environment. Therefore, their strength and nature should be predicted using robust, accurate quantum chemical methods with as few errors as possible.

The easiest way to quantify intermolecular interactions is with a supermolecular approach. Here, improvements have been introduced to rather low-cost methods such as Hartree–Fock (HF),^9−12^ DFT,^13−15^ and Møller–Plesset second order perturbation theory (MP2).^16−18^ However, to produce robust and accurate results, a method such as CCSD(T)^19,20^ (coupled-cluster with single, double, and perturbative triple excitations), which formally scales as *N*^7^, close to the complete basis set limit (CBS), has to be utilized.

With symmetry-adapted perturbation theory (SAPT),^21−25^ interaction energies as a sum of physically meaningful size-extensive components can be computed: electrostatics (els), Pauli repulsion (ex), induction (ind), and dispersion (disp). In contrast to supermolecular approaches, SAPT energies are free of the basis set superposition error (BSSE) by construction. In the framework of SAPT, the zeroth-order Hamiltonian can be considered as a sum of unperturbed noninteracting monomer Fock operators. The whole Hamiltonian also includes the interaction potential and intramonomer correlation effects of each monomer. Provided that the interaction is sufficiently weak, an expansion based on Rayleigh–Schrödinger perturbation theory can give the interaction energy. At first-order, electrostatics and exchange terms appear, which are Coulombic interactions between the monomers with unperturbed electron densities, including interpenetration of charge clouds and a repulsive energy arising from the fermionic antisymmetry requirements of the dimer wave function, respectively. The polarization components at second-order are induction and dispersion. Induction is an attractive effect created due to the response of the charge density of one monomer to the static electric field of another monomer, which also contains charge-transfer effects. Dispersion results from the dynamical electron correlation between the two monomers. Induction and dispersion terms are accompanied by their exchange-repulsion counterparts.

Among several variants of SAPT, DFT-SAPT^26−31^ [sometimes also called SAPT(DFT)^32,33^] has a favorable scaling of the computational demand with system size and acceptable accuracy at second-order. Using density-fitting techniques^34^ to avoid the computation of 4-center electron repulsion integrals, the overall scaling of the method is *N*^5^.

The total interaction energy in DFT-SAPT can be written as follows
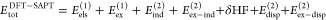
1where *E*_els_^(1)^, *E*_ind_^(1)^, and *E*_disp_^(2)^ are polarization terms, namely electrostatics, induction, and dispersion, respectively, along with their corresponding exchange-repulsion counterparts *E*_ex_^(1)^, *E*_ex-ind_^(2)^, and *E*_ex-disp_^(2)^. The term δHF is defined as
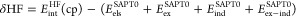
2where *E*_int_^HF^(cp) is the counterpoise-corrected HF interaction energy, which is assumed to consist of electrostatics, exchange-repulsion, and induction (plus its exchange term) components. The remaining terms are the analogous interaction components calculated at the HF-SAPT (SAPT0) level with the same basis set. δHF is an approximation for higher-order induction terms and is thus added to the induction energy.

DFT-SAPT provides a rigorous description of the dispersion energy by means of coupled-perturbed time-dependent DFT (TD-DFT) response functions without causing methodological inconsistencies. Moreover, all polarization terms, including electrostatics, induction, and dispersion, become exact if the exact exchange–correlation (xc) potential and the exact xc-kernel are known and employed.^31^ For example, Kohn–Sham orbitals can be constructed by using exact densities through the ZMP approach in the SAPT framework.^35^

Usually, the intramonomer electron correlation is described through an appropriate asymptotically corrected density functional. The lack of exact exchange contribution in DFT often underestimates the energy differences between the highest occupied molecular orbital (HOMO) and the lowest unoccupied molecular orbital (LUMO), which affects the accuracy of second-order interaction contributions significantly. This problem is ameliorated by using hybrid functionals, including a fraction of exact exchange that is self-interaction-free. When asymptotic corrections are used, problems with the simulation of the xc-kernel frequency dependence for the dynamical response in the dispersion interactions can arise. This is because we are not using the functional derivative (*v*_xc_ = δ*E*_xc_[ρ]/δρ) to generate xc-potentials.^36^ In the case of adiabatic local density approximation (ALDA) and nonhybrid functionals, the xc-kernel *f*_xc_^ALDA^ is computed using *f*_xc_^LDA^, regardless of the actual model of the utilized *v*_xc_ to a good approximation.^37,38^ When the functional is a hybrid functional, the response function magnitude is decreased, thus reducing the corresponding dispersion energy.^32^ To resolve this problem, a hybrid ALDA xc-kernel is used, which consists of a fraction of the nonlocal exact exchange response kernel (*x*) and (1 – *x*)*f*_xc_^ALDA^.^30^ The use of generalized gradient approximation (GGA) instead of LDA in the TD-DFT kernel is not recommended since it reduces the speed of calculations significantly for larger complexes.^32^

In addition to the equilibrium intermonomer separation, some other shorter and longer distances should be taken into account to provide a more realistic and practical description of noncovalent interactions, which can be used, for example, to develop analytic potential energy surfaces (PESs)^39^ and to predict molecular crystal structures^40^ and liquids. The development of reliable intermolecular potential curves separable into physically meaningful contributions is crucial for the production of a new generation of force fields to allow for robust and accurate molecular dynamics simulations.^41,42^

To generate such data, we took advantage of the available data sets developed by Řezáč and co-workers, which cover a large number of dissociation curves for H-bonds in organic molecules (HB375×10^43^), H-bonds including sulfur, phosphorus, and halogens (HB300SPX×10^44^), ionic H-bonds (IHB100×10^43^), σ-hole interactions including chalcogen bonds, halogen bonds, and pnicogen bonds (SH250×10^45^), repulsive contacts (R739×5^46^), π–π interactions, dispersion driven complexes, and different types of interactions found in organic and biomolecules (S66×8^47^), interactions of halogenated molecules (X40×10^48^), and London dispersion in an extended chemical space (D442×10^49^) to create a new set of data for the interaction energy components. The reasons for choosing these data sets are their diversity, extensiveness, and systematic classification based on the type of interactions, the existence of potential curves for each complex, and high-quality CCSD(T)/CBS interaction energies for all the geometries and distances.

In this work, we thus computed the values of the main energy components, namely, electrostatics, exchange-repulsion, induction, and dispersion, for 19,293 dimer structures involving 2312 potential curves, each of which with two hybrid xc-potentials, resulting in 154,344 data points. Out of these calculations, for 5778 structures containing only first-row atoms and H, more expensive calculations were again performed by using a non-approximated expansion in exchange-induction and exchange-dispersion energies. Additionally, for 1000 ionic H-bonds, only induction terms were recalculated. The charge transfer as another important energy component was also calculated for each system in the HB375×10 and HB300SPX×10 data sets. In total, together with total interaction energies, the generated data amounted to 354,106 quantum chemical values categorized into different groups based on the employed methods and/or types of interactions. Chosen parts of these data can be utilized by researchers in the field of semiempirical methods, force field developers, and, especially, machine-learning engineers.

## 2 Computational Details

All geometries were taken from www.nciatlas.org and www.begdb.org without reoptimization for direct comparison with the available CCSD(T) data. For S66×8, HB375×10, HB300SPX×10, R739×5, and D442×10, the Molpro 2020 suite of programs^50^ was used to perform DF-DFT-SAPT (DFT-SAPT with density-fitting approximations) calculations. For the data sets containing σ-hole interactions such as SH250×10 and X40×10, a modified version of Molpro was used to perform regularized SAPT.^51,52^ Due to some convergence issues related to the monomer wave functions of the charged systems in the IHB100×10 data set, the Psi4 program package^53^ was used in lieu of Molpro in this case. SAPT(DFT) was applied to these systems, and whenever normal acceleration methods failed, the quadratic convergence approach was used to converge the energies. Psi4 was also used to calculate higher-order SAPT energies with and without the δMP2 corrections.

B3LYPAC was also examined besides PBE0AC for all of the dimers as the underlying xc-potential. For both, the asymptotic behavior of xc-potential was corrected by means of the gradient-regulated asymptotic correction (GRAC) approach.^54^ The HOMO energy and the ionization potential (IP) of all monomers were calculated at both the PBE0 and B3LYP levels to obtain shift parameters for each functional.

The correlation-consistent basis sets of Dunning and co-workers^55−57^ were used for all the calculations, unless mentioned otherwise. The basis sets were augmented by diffuse functions, which accelerate basis set convergence for NCIs.^58−60^ For the systems without σ-hole interactions, the first row elements (also H and He) were computed by using the aug-cc-pVnZ (n = T, Q) basis sets. For the second row atoms (P, S, Cl, and Ar), the aug-cc-pV(n+d)Z basis sets were employed.^61^ These basis sets lead to improved convergence when extrapolating the dissociation energy to the CBS limit.^61^ For the post-d group elements such as Br and I, augmented correlation consistent basis sets in conjunction with small-core relativistic pseudopotentials (PPs)^62,63^ were utilized to account for the relativistic effects.^64^ As JKFIT or Coulomb and exchange fitting basis sets, def2-AnZVPP/JKFIT (n = T, Q)^65^ and as MP2FIT auxiliary basis sets, similarly, def2-AnZVPP/MP2FIT (n = T, Q)^66^ from the Molpro basis library,^67^ including additional diffuse functions, were used. The remaining elements (H, He, and first and second row atoms) were treated with the aug-cc-pVnZ/JKFIT^68^ and aug-cc-pVnZ/MP2FIT^69^ (n = T, Q) auxiliary basis sets.

Due to the existence of post-d group elements engaged directly in the binding between monomers in the σ-hole interactions, subvalence correlation effects^70^ were taken into account. Therefore, we tried to avoid using the frozen core approximation, and weighted core-valence pseudopotential-based relativistic aug-cc-pwCVnZ-PP (n = T, Q) basis sets were used to describe heavy atoms. For the sake of consistency, all other elements were also described with aug-cc-pwCVnZ-PP (n = T, Q) basis sets in these calculations.

In σ-hole interactions, especially with heavy atoms, where nuclear attraction is so large that electrons freely tunnel there from the other monomer, perturbation expansion theory can break down.^71^ To avoid this charge-transfer effect, a regularized SAPT introduced by Misquitta was utilized.^51,72^ In this approach, a barrier potential is added to the nuclear attraction so that the charge transfer (CT) is almost inhibited, and the remaining contribution to the (exchange-) induction energy is as usual obtained by computing the δHF term. The regularization parameter η was chosen to be 3.0 au—which is used to obtain the nuclear screening length scale, η^–1/2^—as recommended by Misquitta.^51^

For the data sets HB375×10 and HB300SPX×10, the charge transfer energies were calculated with the PBE0AC potential and the aug-cc-pVQZ basis set. The CT energy is defined as follows
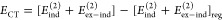
3where subscript reg indicates that the energy terms in brackets result from a regularized SAPT calculation, as described above. It should be mentioned that the reported CT energies in this study are not to be canceled out by the exchange-induction energy, as this factor has already been taken into account.

The complete basis set limit was calculated by extrapolating the *E*_disp_ and *E*_ex-disp_ values using two energy points (T, Q), for which the standard formula X^–3^ introduced by Halkier and co-workers was utilized.^73^ The extrapolation of the remaining components, such as induction, electrostatics, and exchange-repulsion, is not required since these terms converge more rapidly with the size of the basis set.^74^ The aug-cc-pVQZ basis set was considered a sufficiently large basis set for these terms.

All the calculations were automated through an interface to Molpro implemented in a local version of Atomic Simulation Environment (ASE)^75^ by us.

In order to analyze percentage errors, we used the mean capped unsigned relative error (MCURE)^76^ as used by Patkowski and co-workers. It is especially suitable for interaction energies along the dissociation curves. The details of this approach are given in the Supporting Information.

## 3 Results and Discussion

In Table 1, we provide a summary of the compositions of all data sets and their subsets used in this work. Detailed information about the compounds involved in each subset can be found in the references related to the data sets (vide supra). Here, we used similar classifications based on the type of interactions, considering the dimers available in all data sets. In total, 35 categories representing different types of interactions were built. Some of these categories, excluding those related to R739×5 and S66×8, were then merged, resulting in 21 categories (see Section 3.2). The Disp(π–π) category introduced in this paper consists of the systems with π–π interactions chosen from the data sets S66×8, D442×10, and X40×10 (Table S2).

**Table 1:** Amount of Complexes (Potential Curves) in Each Subset of the Original Data Sets

data set	subset	number	data set	subset	number
HB375×10	OH···N	45	S66×8	OH···N	5
	OH···O	60		OH···O	9^a^
	NH···N	53		NH···N	1
	NH···O	65		NH···O	9
	CH···N	20		Disp(π–π)	10
	CH···O	19		Disp(HCNO)	13
	noHB	113		Mix	20
					
D442×10	Disp(HBCNO)	105	X40×10	OH···O	2
	Disp(PS)	103		AH···O	4
	Disp(Halogen)	94		AH···N	2
	Disp(NobleGas)	140		AH···F	1
	Disp(π–π)^b^	64		AH···Cl	1
				Disp(π–π)	2
IHB100×10	IHB(Cation)	35		Disp(Halogen)	4
	IHB(Anion)	65		Mix	6
				σ-hole(X–π)	4
SH250×10	σ-hole(Cl)	29		σ-hole(Cl)	4
	σ-hole(Br)	36		σ-hole(Br)	5
	σ-hole(I)	42		σ-hole(I)	5
	σ-hole(S)	31			
	σ-hole(Se)	44	HB300SPX×10	AH···F^c^	41
	σ-hole(P)	33		AH···Cl	32
	σ-hole(As)	35		AH···Br	17
				AH···I	19
R739×5	Rep(HBCNO)	170		AH···S	54
	Rep(PS)	154		AH···P	52
	Rep(Halogen)	235		AH···N	34
	Rep(NobleGas)	180		AH···O	51

a One of these complexes has also an NH···O interaction.

b This category overlaps with the other subsets of D442×10 (Table S2).

c A in AH···B is C, N, O, P, S, F, Cl, Br, or I (C, N, and O do not exist as A when B is N or O).

### 3.1 Accuracy of DFT-SAPT

The total interaction energies calculated with eq 1 were compared to the CCSD(T)/CBS values supplied in the references related to the data sets (vide supra) to assess the performance of DFT-SAPT or SAPT(DFT) along the dissociation curves. For the X40×10 and S66×8 data sets, the latest revisited data were extracted from the literature.^70,77^

#### 3.1.1 Single Exchange Approximation and B3LYPAC/PBE0AC xc-Potentials

The exchange energy components in SAPT are expanded in powers of the intermolecular overlap*(S)* often truncated after the first terms, which is called the single-exchange (*S*^2^) approximation. This may cause the exchange interaction energies to become inaccurate when monomers are very close to each other.^78,79^

Normally, the first-order *E*_ex_^(1)^ values obtained from an infinite expansion in *S* (*S*^∞^) are included in the total interaction energy^80^ and also in the calculation of δHF. However, *S*^∞^ is not commonly used for the induction and dispersion parts because it was not formulated in the early stages of SAPT^79^ and is also computationally much more demanding.

The effect of the single-exchange approximation used in the *E*_ex-ind_^(2)^ and *E*_ex-disp_^(2)^ terms for HB375×10, S66×8, and those D442×10 systems containing only light atoms (HBCNO) was analyzed. This effect was also investigated in the *E*_ex-ind_^(2)^ term for the IHB100×10 data set. The contribution of *E*_ex-disp_^(2)^ to the total energy is not significant, especially for systems with a high induction-to-dispersion (ind.disp^–1^) ratio such as ionic H-bonds.^81^

In the present paper, the symbol *S*^2^ refers to the calculations in which exchange-induction and exchange-dispersion were treated using the single-exchange approximation, while *S*^∞^ means that these exchange components were obtained from an infinite expansion in the intermolecular overlap (for the systems of the IHB100×10 data set, the dispersion-exchange term was only calculated with the *S*^2^ approximation). The first-order exchange-repulsion energy was always calculated using *S*^∞^, even when the symbol *S*^2^ is used in the related method.

As shown in Table 2, in many cases, *S*^∞^ surprisingly increases the RMSE values. Only for the HB375×10 data set, using PBE0AC, a noticeable improvement is observed, particularly for the typical H-bonded systems (HB) at short intermonomer distances. For the IHB100×10 data set, PBE0AC with the *S*^2^ approximation shows the best performance for 75% of the dissociation curves.

**Table 2:** RMSE Values (kJ mol^–1^) of the SAPT Calculations with the Asymptotically Corrected B3LYP and PBE0 Potentials Using *S*^2^ and *S*^∞^ Classified Based on the Short Range (s), Longer Range (l), Equilibrium (e), and All Distances (a) for the Dimers without Heavy Atoms^a^

	IHB100×10			S66×8+D442×10(HBCNO)				HB375×10			
method/range	All	C^b^	A	All	HB^c^	Disp	Mix	All	HB	CHX^d^	noHB
PBE0AC(*S*^2^)/a	6.3	5.1	6.8	0.9	1.0	0.9	0.5	1.7	1.9	1.3	1.6
PBE0AC(*S*^∞^)/a	6.8	5.4	7.4	0.9	1.3	0.9	0.5	1.6	1.6	1.2	1.6
B3LYPAC(*S*^2^)/a	7.9	7.0	8.3	1.2	2.6	0.9	0.6	1.7	2.1	0.8	1.1
B3LYPAC(*S*^∞^)/a	9.6	8.0	10.3	1.4	3.1	0.9	0.6	2.1	2.6	0.9	1.1
PBE0AC(*S*^2^)/e	6.8	5.9	7.2	0.8	1.4	0.7	0.5	1.1	1.2	0.7	0.9
PBE0AC(*S*^∞^)/e	7.5	6.4	8.1	0.8	1.7	0.6	0.5	1.2	1.3	0.7	0.9
B3LYPAC(*S*^2^)/e	8.8	8.3	9.1	1.4	3.3	0.7	0.6	1.9	2.4	0.8	0.8
B3LYPAC(*S*^∞^)/e	10.1	9.0	10.6	1.5	3.8	0.7	0.7	2.2	2.7	0.8	0.8
PBE0AC(*S*^2^)/l	5.3	4.7	5.6	0.5	1.0	0.4	0.3	0.7	0.9	0.5	0.5
PBE0AC(*S*^∞^)/l	5.6	4.9	6.0	0.5	1.1	0.3	0.3	0.8	0.9	0.5	0.5
B3LYPAC(*S*^2^)/l	6.2	6.0	6.3	0.8	2.0	0.4	0.4	1.2	1.6	0.5	0.5
B3LYPAC(*S*^∞^)/l	6.8	6.3	7.1	0.9	2.3	0.4	0.4	1.4	1.7	0.5	0.5
PBE0AC(*S*^2^)/s	7.5	5.7	8.3	1.3	1.1	1.3	0.9	2.6	2.8	2.0	2.4
PBE0AC(*S*^∞^)/s	8.2	6.2	9.2	1.4	1.7	1.4	0.9	2.3	2.3	1.8	2.4
B3LYPAC(*S*^2^)/s	9.9	8.4	10.6	1.7	3.9	1.3	0.9	2.3	2.7	1.0	1.6
B3LYPAC(*S*^∞^)/s	12.6	10.1	13.8	1.9	4.9	1.4	1.0	2.9	3.5	1.2	1.7
nPBE0AC(*S*^2^)/a	75	22	53	59	15	42	2	60	49	4	7
nPBE0AC(*S*^∞^)/a	21	10	11	36	7	25	4	118	96	6	16
nB3LYPAC(*S*^2^)/a	3	2	1	59	1	44	14	141	64	18	59
nB3LYPAC(*S*^∞^)/a	1	1	0	17	0	17	0	56	14	11	31

a For each category, the number of complexes (*n*), for which the corresponding method shows the lowest RMSE is given in the last four rows.

b C: IHB(Cation), A: IHB(Anion).

c Typical H-bonds (NH···O, OH···O, OH···N, and NH···N).

d CH···N and CH···O.

PBE0AC(*S*^2^) shows the lowest RMSE values for the S66×8+D442×10(HBCNO) dimers in general, as given in Table 2. However, considering the total number of cases in which a good performance of PBE0AC(*S*^2^) is observed (59 dimer dissociation curves), B3LYPAC(*S*^2^) shows similarly good results. For the mixed-influence and dispersion-dominated systems, B3LYPAC(*S*^2^) is the best method for most of the dimers. Here, *S*^2^ decreases the RMSE values compared to *S*^∞^, especially at short distances.

Given all of the systems in HB375×10, B3LYPAC(*S*^2^) yields the lowest RMSEs for 141 curves, mainly because of the systems with weak interactions. For these systems, the overall ind.disp^–1^ ratios were observed to be small compared to those of typical H-bonds and to those of IHB100×10 systems.

**Figure 1:**
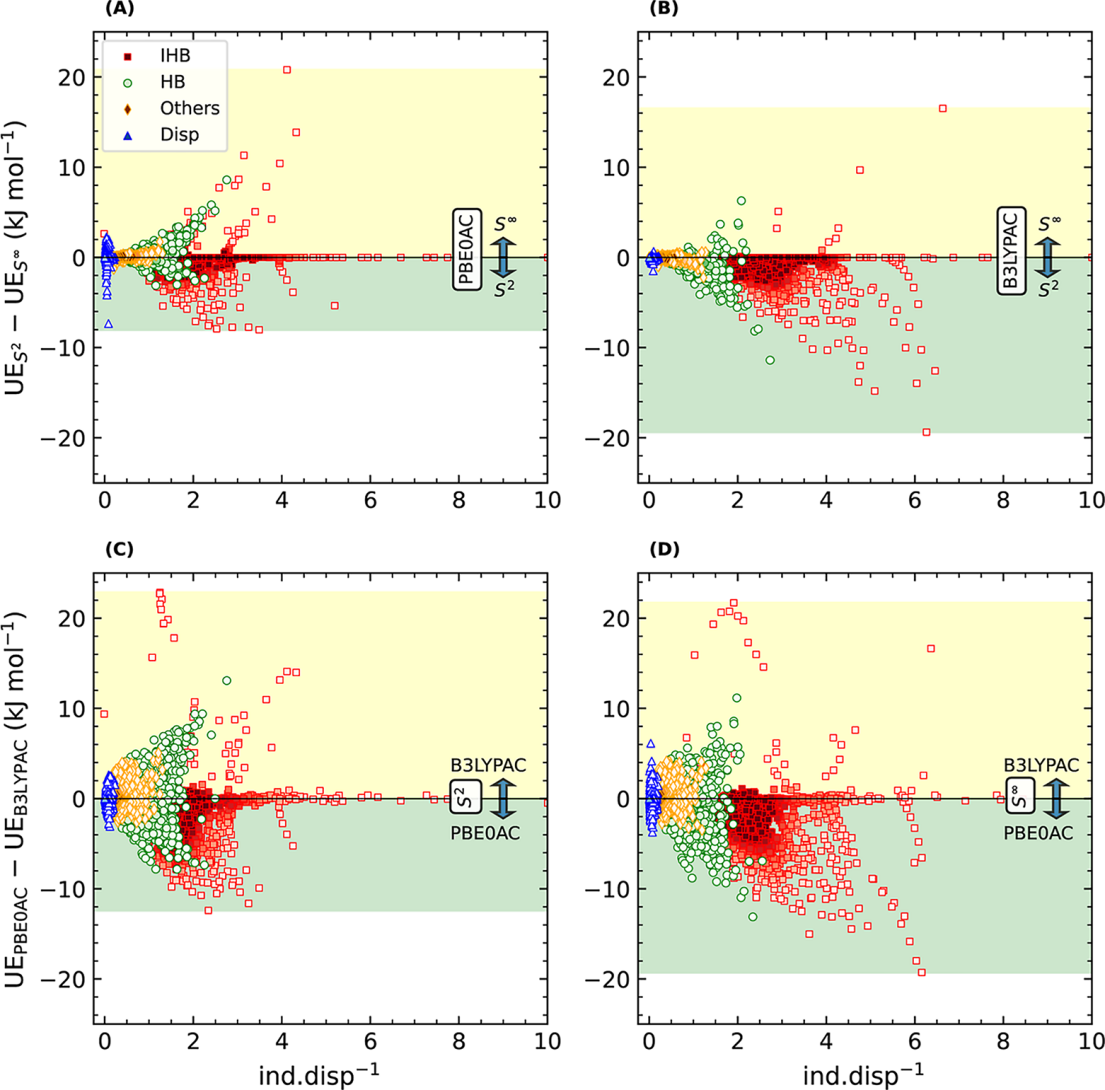
Distribution of unsigned error (UE) differences vs induction to dispersion (ind.disp^–1^) ratios for the SAPT calculations of the data sets IHB100×10, HB375×10, and S66×8+D442×10(HBCNO) categorized based on the type of interaction (IHB: ionic H-bonds, HB: typical H-bonds, Disp: dispersion-dominated, and Others: mixed-influence complexes (Mix), CHX, and noHB). The error difference was calculated as UE_lower_ – UE_upper_, where UE_lower_ is the unsigned error related to the method denoted on the lower area (green shaded area below zero) and vice versa. Therefore, for the data points shown on the lower area, the lower method produces more accurate results compared to the upper ones (yellow shaded area), and vice versa. The shaded areas also show the range of the data distribution. (A,B) comparison of *S*^2^ with *S*^∞^. (C,D) effect of fixed *S* and xc-potentials compared. The densities were analyzed using the KDE approach once for each category and once for all of the data points (Supporting Information).

In Figure 1, each method is compared with its counterpart based on the difference between their related unsigned errors for each energy single point of the three data sets mentioned in Table 2 in terms of ind.disp^–1^ ratios. The distribution of the data is also classified according to the type of interaction. The error difference along the vertical axes is obtained by subtracting the unsigned error of the method with the symbol represented on the lower area (under zero) from the unsigned error of that on the upper area. Therefore, if the resulting error difference is negative, it means that the lower method shows a smaller error and, hence, is more accurate than the upper one for a given point. Likewise, the positive values imply the superiority of the upper method over its counterpart.

At first glance, we observe that with increasing the ind.disp^–1^ ratio, the sensitivity (the range of error differences) of the results to changing the underlying methods (xc-potentials and the *S*^2^ approximation) of SAPT increases by and large. The whole spans of lower and upper data distributions are depicted with yellow and green shaded areas, respectively. The largest differences are associated with the ionic H-bonds, whereas the dispersion-dominated (Disp) complexes, together with Others, exhibit relatively small differences. Typical H-bonds are mainly between these two extremes.

Figure 1A depicts a very detailed comparison between *S*^2^ and *S*^∞^ once the xc-potential is PBE0AC. Most of the IHB and HB individual dimers are more accurately computed using the *S*^2^ approximation. The exact number of data points in the negative and positive areas is given in Table 3 (the percentage differences are shown in Figure S4). For the IHB100×10 data set, more than 70% of the dimers exhibit a lower error with *S*^2^. For the groups Disp and Others, *S*^∞^ works slightly better than *S*^2^. However, the total percentage difference is around 12% in favor of *S*^2^, considering all dimers of the four groups.

**Table 3:** Number of Positive and Negative Values and Zero if Any (Unsigned Error Differences) in Figure 1 for Each Subplot [(A), (B), (C), and (D)]

	(A)			(B)			(C)		(D)	
category	(+)	(−)	(0)	(+)	(−)	(0)	(+)	(−)	(+)	(−)
IHB	137	863	0	63	937	0	161	839	136	864
HB	1086	1328	0	497	1916	1	868	1546	772	1642
Disp	617	607	10	500	724	10	617	617	527	707
Others	951	729	0	800	872	8	1166	514	1137	543
All	2791	3527	10	1860	4449	19	2812	3516	2572	3756

Once the xc-potential is B3LYPAC, the *S*^2^ approximation is the best for all types of interactions (Figure 1B, Table 3). Here, the difference is close to 90% in favor of *S*^2^ for the IHB100×10 data set and around 41% when taking all four groups into account. Therefore, for B3LYPAC, the advantage of the *S*^2^ approximation is much more noticeable compared to that of PBE0AC. This can also be deduced from the results in Table 2.

Figure 1D and Table 3 show that for most of the systems, the unsigned error arising from B3LYPAC is larger than that from PBE0AC when using *S*^∞^. Only the systems of the Others group are mainly more accurately treated with B3LYPAC(*S*^∞^).

The negative range (green shaded area) in Figure 1C shrinks compared to Figure 1D. Smaller error differences for IHB, HB, and Others are seen in this area. The number of systems with a negative error difference also declines (Table 3) for all groups except for Disp, which remains unchanged. Therefore, the gap between B3LYPAC and PBE0AC decreases when using *S*^2^ rather than *S*^∞^, and this occurs primarily through a reduction in the error of B3LYPAC.

According to the above-mentioned observations, the effectiveness of an infinite expansion in *S* changes between the two xc-potentials, and for B3LYPAC, the use of the approximation gives rise to prominently better results. These findings imply that there is probably an error cancellation between the approximation used in the exchange-induction (-dispersion) energy expansion and the xc-potential used. Using SAPT interaction energies without the δHF term, we reached a similar outcome with even larger errors for *S*^∞^ compared to *S*^2^ (Figure S3).

Considering unsigned errors and RMSEs, for the calculation of weak H-bonds and dispersion-dominated systems, B3LYPAC is preferred to PBE0AC in the SAPT framework. However, for typical and ionic H-bonds, PBE0AC is still recommended as the best underlying xc-potential. The single-exchange approximation that is used frequently for induction and dispersion components, can improve the accuracy of the results through error cancellation. The degree of this cancellation is very significant when the xc-potential is B3LYPAC. Taking this point into account and also due to the relatively high computational costs of *S*^∞^ for the systems containing heavy atoms, all calculations from here on are with the *S*^2^ approximation.

#### 3.1.2 Nature of Interactions and Chemical Elements

**Figure 2:**
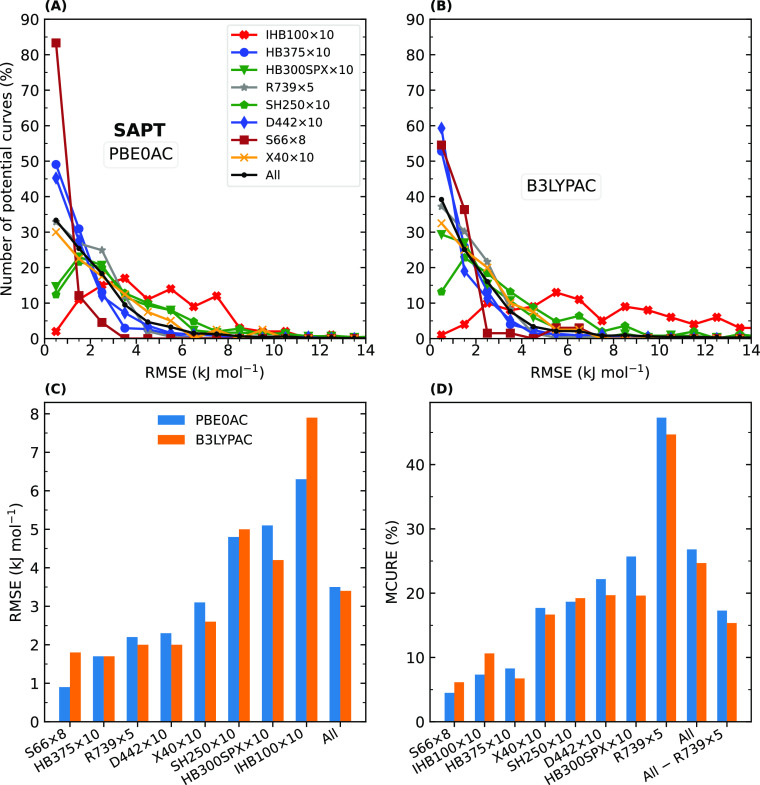
(A,B) Number of dimer potential curves (as a percentage of the total number in the related data set) in each RMSE range for all of the data sets using (A) PBE0AC and (B) B3LYPAC xc-potentials. Each RMSE (calculated for the points of a potential curve) range has a width of 1 kJ mol^–1^, and thus each data point has an uncertainty of ±0.5 kJ mol^–1^. The curves with the same color show similar behaviors. (C) Total RMSE values for each data set with different xc-potentials. (D) Mean capped unsigned relative errors (MCUREs). The last column shows the MCUREs for all of the data, excluding the R739×5 data set.

The primary classification based on the original data sets can reflect a general outlook on the performance of DFT-SAPT in analyzing different noncovalent bonds. In Figure 2A,B, the histograms of the RMSE of each potential curve calculated for each dimer system using the two xc-potentials are displayed. The lines with the same color are related to the data sets with similar trends. In the case of PBE0AC, the patterns of SH250×10 and HB300SPX×10 are very similar. Moreover, the variation of the X40×10 plot at some points bears a close resemblance to that of those two data sets. The similarities are also seen when using B3LYPAC. SH250×10 and HB300SPX×10 have particular atoms such as halogens, P, and S in common, and X40×10 contains halogens but no S and P atoms. Although H-bonds and σ-hole interactions can be significantly dissimilar (for example, the histograms, total RMSEs, and MCUREs (Figure 2C,D) for HB375×10 and SH250×10 are completely different), their errors become much closer to each other when the same types of elements are involved (HB300SPX×10 and SH250×10).

Moreover, in spite of a similarity between D442×10 and HB375×10 in Figure 2A–C, their percentage errors shown in Figure 2D are noticeably different. The data sets containing heavier atoms, by and large, exhibit around 10% larger percentage errors compared to the data sets of only light atoms.

**Figure 3:**
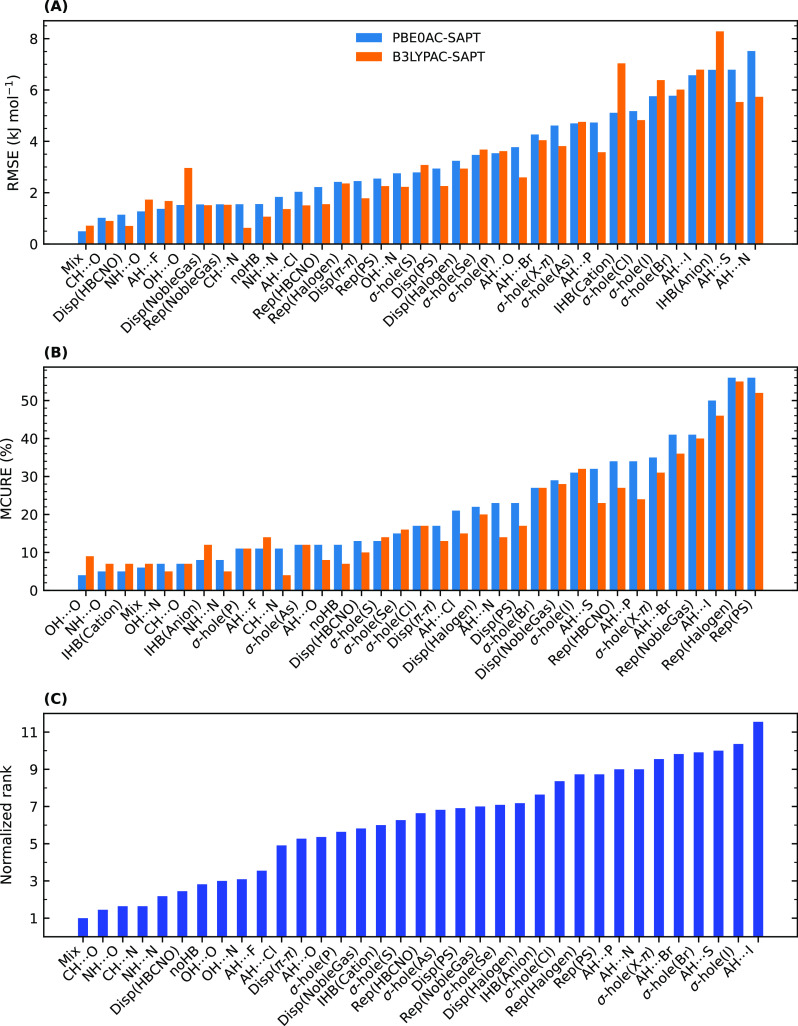
(A) Total RMSE values for different types of interactions are categorized by including all the systems of the data sets and sorted based on the values related to PBE0AC. (B) Mean capped unsigned relative errors for all of the categories. (C) Categories ranked according to their related RMSEs and MCUREs, considering both xc-potentials.

In Figure 3A,B, the systems are categorized based on the type of their noncovalent interactions, and their related RMSE and MCURE with the PBE0AC and B3LYPAC xc-potentials are shown. The categories were chosen in accordance with Table 1. In the case of light atoms, B3LYPAC increases the errors (both RMSE and MCURE) for mixed-influence, NH···O, and especially OH···O interactions, while for other interactions, such as OH···N, NH···N, weak H-bonds (CH···N, CH···O, noHB), repulsive contacts, and π–π interactions, it leads to more accurate interaction energies. For the ionic H-bonds, PBE0AC seems to outperform B3LYPAC, as discussed in Section 3.1.1. It is also obvious in Figure 2A,B that IHB100×10 has a completely different histogram compared to other data sets, especially when the xc-potential is B3LYPAC. Nearly zero percentages for the errors below 1 kJ mol^–1^ and considerable numbers around 10% are observed for large RMSEs. B3LYPAC increases the population of systems with RMSE values larger than 8 kJ mol^–1^. However, large RMSEs for such strong interactions can be acceptable, and accordingly, their relative errors are similar to those of routine H-bonds. To display the differences between various categories, we ranked them by taking equally both measures of error related to both xc-potentials into account (Figure 3C). The results revealed that, in general, interactions containing heavy atoms such as Br and I have the worst ranks, while typical H-bonds with first-row atoms are the best systems for DFT-SAPT.

When investigating the features in the systems such as σ-hole interactions and H-bonds with heavier atoms causing these large deviations, induction appears to be the most challenging energy component for the DFT-SAPT method, as the addition of δHF is really crucial to reaching dissociation curves close to the CCSD(T)/CBS ones. Considering the role of induction energy in neutral noncovalent bonds, dispersion-dominated and σ-hole interactions constitute two extremes. For example, as given in Table S7, the average induction energy for the D442×10 systems is −1.64 kJ mol^–1^, while this value for the case of SH250×10 after regularization is −10.41 kJ mol^–1^ (these values are reported without the δHF correction). In fact, the amount of induction energy in many SH250×10 systems is so high that the second-order perturbation expansion will break down if the nuclear potential is not regularized, and consequently, unrealistically large energies will be obtained. However, this catastrophe is not observed in typical or ionic H-bonds consisting of light atoms, despite a large amount of induction. This component still consists of two terms, namely, polarization and charge transfer (CT) energies. Although there are no robust ways to calculate the CT term, according to an approach introduced by Misquitta,^51^ an amount of this energy can, depending on the degree of regularization, be extracted from the induction energy. This approach is not appropriate to almost all of the SH250×10 systems, for which nonregularized energies become unrealistic. This is also the case for many systems in X40×10. On the other hand, it is predicted for D442×10 that this CT energy will be a small portion of its induction energy, which is on average −1.64 kJ mol^–1^. Given these points and to draw a rational comparison, we used this approach to capture CT energies in the complexes of the HB375×10 and HB300SPX×10 data sets (many of the systems in S66 can also be found in HB375 with small differences in their geometries).

**Figure 4:**
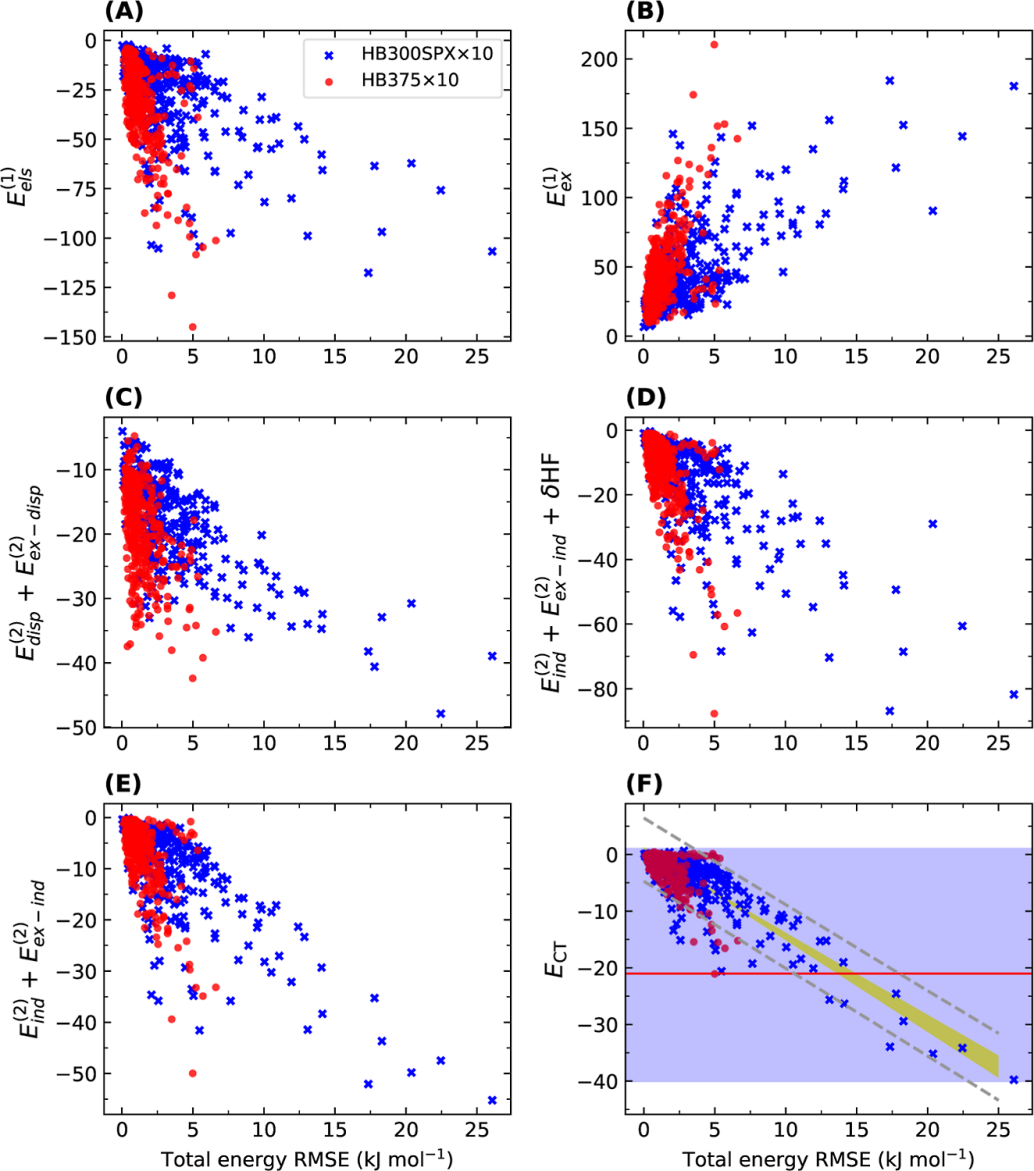
Average of different PBE0AC-SAPT components and the average of charge transfer (CT) vs the RMSE of the total interaction energy for each dissociation curve of the HB375×10 and HB300SPX×10 data sets. The blue shaded area in (F) shows the range of CT energies for HB300SPX×10, and the red line indicates the largest CT found in HB375×10. All energy and error values are in kJ mol^–1^.

Figure 4 provides helpful information to investigate the source of errors in many DFT-SAPT calculations. It shows how energy components for a dissociation curve, on average, vary when the RMSE value of the corresponding total interaction energy increases. The scattering patterns of electrostatics and exchange components are very similar but with different signs (Figure 4A,B). Although these two terms for HB375×10 and HB300SPX×10 vary in a similar range, the RMSEs associated with the latter become much higher with increasing the strength of interaction. The same is true for dispersion, induction, and induction with δHF (Figure 4C–E). The RMSE values of HB375×10 are mostly less than 5 kJ mol^–1^, whereas a significant deviation is observed for the HB300SPX×10 case. For all of the above-mentioned terms, however, the energies vary within similar ranges. These plots imply that there must be another phenomenon in the HB300SPX×10 systems causing these large deviations, which is not that prominent in HB375×10. The scattering plot of the average CT energies against the RMSE values underpins this assumption (Figure 4F). All systems with large RMSE values also have large amounts of CT up to −40 kJ mol^–1^, while in the case of HB375×10 whose RMSEs are low, the amounts of CT are nearly less than −20 kJ mol^–1^. This additional CT in HB300SPX×10 is a significantly different observation from the energy decomposition point of view when the ranges of variations over the RMSE values are considered. When relative errors (MCUREs) are plotted vs the mean charge transfer percentages, the adverse effect of CT on the accuracy of the DFT-SAPT results is even better enunciated (Figure S5). Although the degree of linearity declines, the difference between the two data sets and the number of points with a high CT percentage and large errors remarkably increase in the case of relative values. The data points in the HB300SPX×10 set possessing CT energies out of the range of HB375×10 are also of huge MCURE and E_CT_ percentages. These systems are mostly a dimer of one hydrogen halide like HBr or HI and one strong base like ammonia, in which the transfer of an electron is expected when the intermonomer separation decreases, and a proton transfer can also occur.

**Figure 5:**
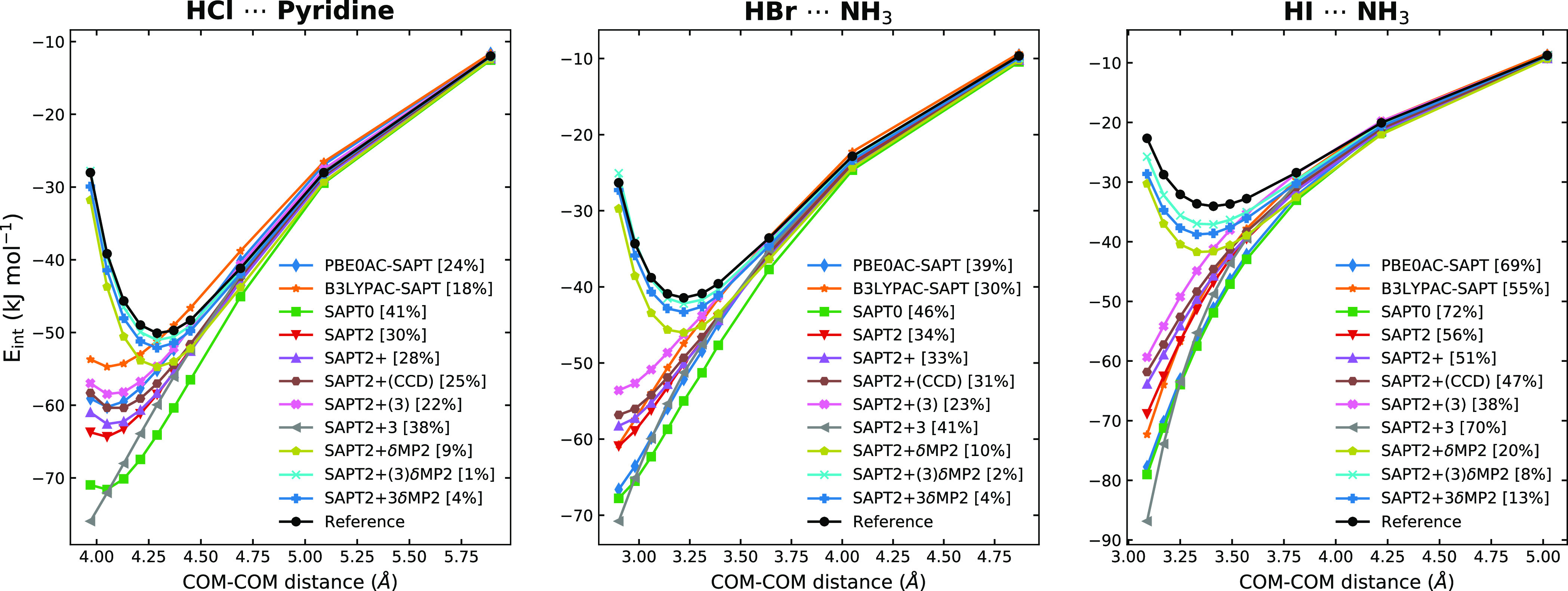
Some problematic complexes from the AH···N category with high CT are used to make comparisons of different SAPT variants. The basis set used for every method except for DFT-SAPT/CBS and the reference [CCSD(T)/CBS] is def2-QZVPPD. In brackets, the percentage errors are shown.

The tunneling of electrons that happens easily into heavier atoms (directly involved in the interaction, especially in close proximity, where the covalent nature of the interaction largely increases) plays a pivotal role in the failure of the SAPT computations in many cases. The comparison of different variants of SAPT^82^ (Figure 5) reveals that even the inclusion of higher-order energies (using, for example, SAPT2+3) cannot cure the problem. Here, a δMP2 term is needed to prevent large deviations. δMP2 is the difference between the correlation part of the counterpoise-corrected MP2 interaction energy [*E*_int_^MP2,corr^(cp)] and higher-order SAPT components as follows^82^
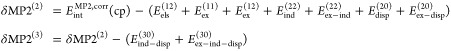
4

The first and second superscripts in the SAPT energy components indicate the order of the perturbative expansion with respect to the intermolecular potential and intramolecular electron correlation, respectively. The *E*_ind-disp_^(30)^ and *E*_ex-ind-disp_^(30)^ terms appear due to the induction-dispersion coupling at third-order. Thus, δMP2^(3)^ is applied to SAPT2+3 energies, while δMP2^(2)^ can be added when SAPT2+ or SAPT2+(3) is used.

Moreover, Figure 5 shows that we cannot attribute this deficiency to the underlying DFT methods since even SAPT based on coupled-cluster doubles (CCD) exhibits a large deviation from the reference curve for these systems when no δMP2 is added. As our calculations on 24 complexes revealed, for these problematic systems, only SAPT2+3 and SAPT2+(3) with δMP2 lead to good results (Supporting Information). A deeper insight into the effects of δMP2 terms leading to these dramatic improvements necessitates further studies, which are beyond the scope of the current work.

AH···F and AH···Cl interactions do not lead to significant CT effects even with heavier atoms (Table S7), and thus, no considerable errors are expected. In addition, with increasing the size of the halogen atom, as the H-bond acceptor, the CT effect is enhanced and larger errors are observed. According to Table S7, the largest mean CT percentage is interestingly related to the AH···I interactions (86%), corresponding to the worst accuracy compared to other heavy-atom-containing H-bonds, as shown in Figure 3B. For most of the AH···B interactions, B3LYPAC mitigates the problem of high RMSEs, except for AH···I and AH···F (Figure 3A). Significant improvement (more than 1 kJ mol^–1^) is observed for AH···N and AH···S interactions, which yield the most inaccurate results when using PBE0AC. Similar results are observed when MCUREs are compared.

#### 3.1.3 Intermonomer-Separations

**Figure 6:**
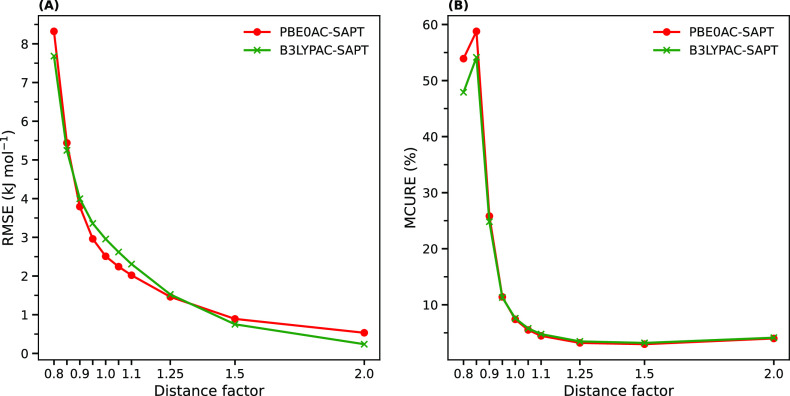
Total RMSEs and total MCUREs vs distance factors (intermonomer separations). The B3LYPAC-SAPT energies at the nonequilibrium distances, especially in short ranges, are slightly more accurate. R739×5 and S66×8 were excluded here because they are not consistent with other 10-point dissociation curves.

It is expected that with decreasing the distance between two monomers, a larger error is observed in the SAPT results due to a larger perturbation and, consequently, a slower convergence of the perturbation expansion. Figure 6 shows that this is true for both xc-potentials in general, as RMSEs can become larger than 7 kJ mol^–1^ at very short separations. However, for some interactions, a drop in the RMSEs at smaller distances can be observed. This can cause nonsmooth trends, as shown in the insets of Figure S8A,B. Due to a sudden energy rise at the smallest distance, a drop in the corresponding relative error occurs, which is obvious in Figure 6B.

Figure 6 also reveals that B3LYPAC exhibits better performance for distances far from the equilibrium point, including short and longer ranges, while for distances close to equilibrium, the PBE0AC-SAPT results can totally be of higher accuracy. This explains why B3LYPAC outperforms PBE0AC for the systems in the R739×10 data set containing only out-of-equilibrium structures, but the reverse is true for the S66×8 data set, where the relative number of nonequilibrium points decreases (Figure 2C).

**Figure 7:**
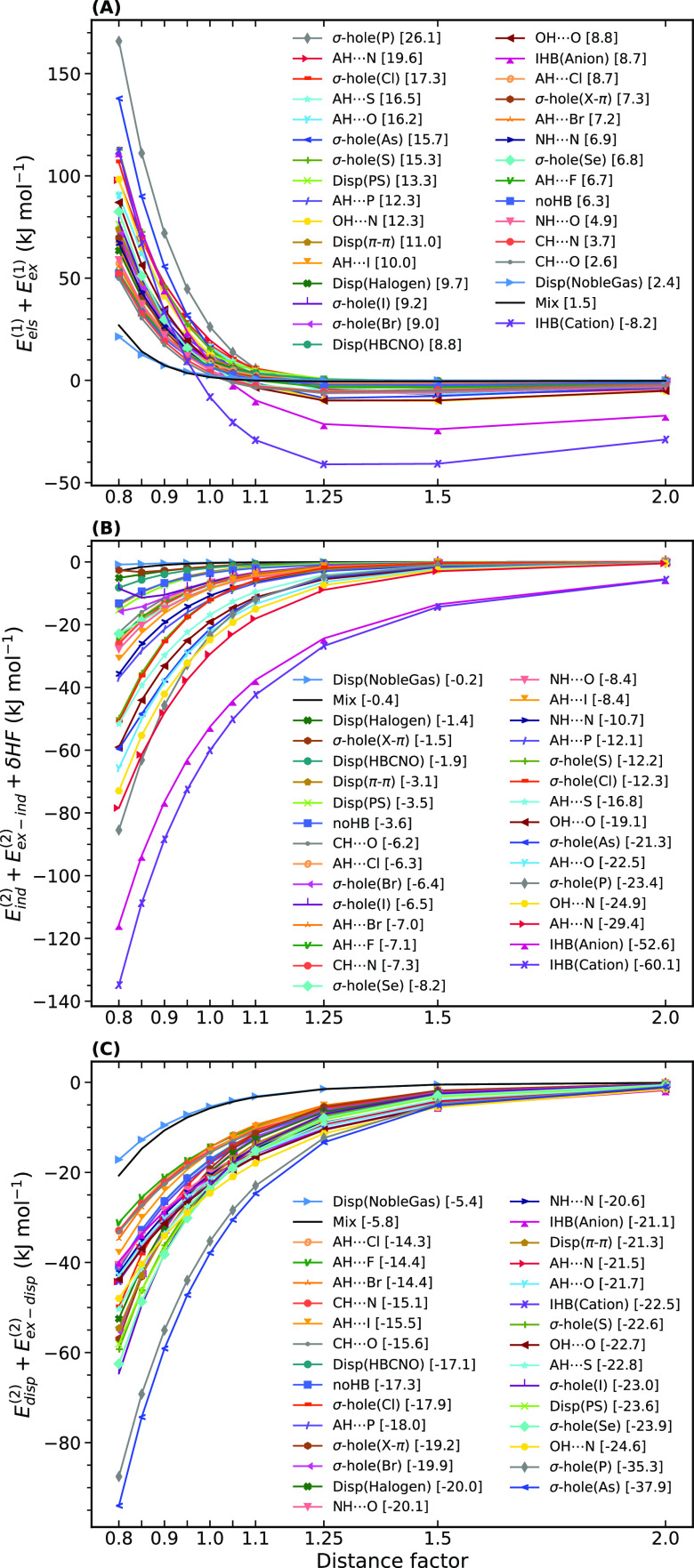
Average of energy components (with PBE0AC) vs intermonomer separation. The values in brackets are the average of the corresponding terms at the equilibrium point, and all the items in the legends are sorted based on these values.

In Figure 7, the average of every stabilizing component plus its related exchange-repulsion term for each category at different intermonomer separations is shown. Electrostatics is severely dominated by the first-order exchange-repulsion energy in short ranges (Figure 7A). Moreover, as shown in brackets in the legends, the sum of *E*_els_^(1)^ and *E*_ex_^(1)^ is a positive value at the equilibrium point, except for the cationic H-bonds being only −8.2 kJ mol^–1^. At longer distances, the attractive electrostatic effects can be stronger than the repulsive forces of the exchange term, but only for the charged dimers can they lead to significant stabilization. This emphasizes how critical the role of induction and dispersion is in the formation of a stable noncovalent bond. For example, despite the long-lasting belief that typical H-bonds are dominated by electrostatic interactions, they exhibit a significant contribution of polarization components and dispersion.^83^

An interesting point revealed by Figure 7A,B (the average of the equilibrium values in brackets) is the inability of induction to neutralize the repulsive effect of the first-order exchange for a variety of interactions even after electrostatics has been included. It implies that for these systems, the formation of a stable noncovalent bond is not possible without a considerable amount of dispersion energy. In addition to dispersion-dominated systems (the Disp category) and weak interactions (noHB+Mix), several groups of σ-hole interactions, such as all the halogen bonds and some H-bonds with heavier atoms, e.g., AH···Cl, Br, and I certainly require dispersion. In contrast, the ionic H-bonds could have a strong binding merely owing to the induction contribution. Routine H-bonds such as NH···O and OH···O could also easily survive without dispersion, but definitely not as strong as they actually are. The ionic H-bonds are the fastest, while the dimers containing rare gases are the slowest in terms of their induction decay with the distance factors. Among the routine H-bonds, the induction energy of OH···N and OH···O interactions decays rather fast with the factor of the equilibrium distance.

Pnictogen bonds exhibit the largest mean value of dispersion at the equilibrium point and also very fast changes with the distance factor (Figure 7C). σ-hole(P) definitely needs this large amount of dispersion because otherwise it cannot exist in its stable form due to its huge exchange-repulsion, which is obvious in Figure 7A.

The OH···N interaction also has significant dispersion. This H-bond (also OH···O to some extent) can be regarded as an intriguingly pronounced form of interaction composed of only light atoms but with large negative energies of induction, charge transfer, and dispersion, thanks to the perfectly coupled H donor and acceptor moieties.

The results obtained from B3LYPAC-SAPT regarding the change of different energy terms with distance factors are very similar to those obtained from PBE0AC-SAPT (Figure S10). The decay rate of the dispersion energy with the COM–COM distances will be later discussed in detail.

#### 3.1.4 Unsigned Errors vs Total Interaction Energies

**Figure 8:**
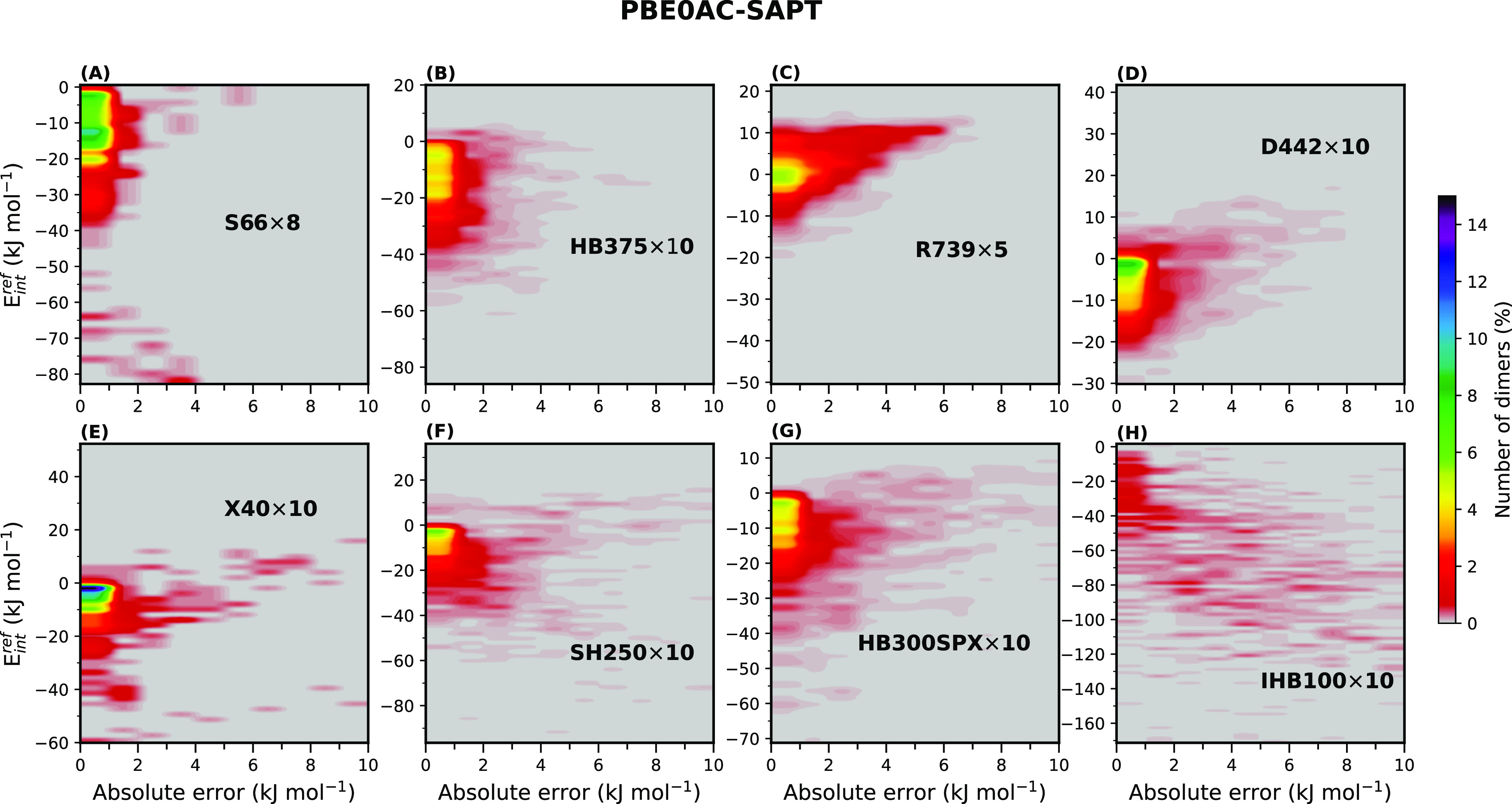
Percentage amount of individual dimers with a particular interaction energy (reference values) and an unsigned error (absolute error) value for their corresponding PBE0AC-SAPT total energy. The bin widths for the data along the *x* and *y* axes are 1 and 2 kJ mol^–1^, respectively. The unsigned errors larger than 10 kJ mol^–1^ were not displayed, but the range of interaction energies is the same as the whole range for each data set.

Figure 8 shows the accuracy of the PBE0AC-SAPT energy in terms of the value of the reference total interaction energy for each data set. Each interaction energy and each unsigned error value have, at most, uncertainties of 2 and 1 kJ mol^–1^, respectively, which are considered the bin widths when calculating the related histograms. Thereby, we define the relative population of dimers as the number of dimers (%) on each 2 × 1 rectangular area.

One common feature for all of the data sets (except for IHB100×10) is that the relative population of the systems with unsigned errors less than 1 kJ mol^–1^ is far higher than that of the systems with larger errors, and generally, as the interaction energy approaches zero, higher populations are observed. Nevertheless, finding a lower error for larger magnitudes of interaction energy is of more interest because, first, it implies a more negligible inaccuracy, and second, it is somewhat surprising that SAPT has a theory based on small perturbations and describes strong interactions very well.

As shown in Figure 8A, most of the S66×8 dimers are distributed in the interaction energy range of 0 to −40 kJ mol^–1^ with a high probability of finding an error less than 1 kJ mol^–1^. This range can be again divided into two nearly equal subranges: the first from 0 to −20 kJ mol^–1^ and the second from −20 to −40 kJ mol^–1^, with the latter showing lower populations. A similar situation, by and large, is present for HB375×10, X40×10, SH250×10, and HB300SPX×10 (Figure 8B,E–G). In the case of D442×10 (Figure 8D), only the first subrange of interaction energies (between 0 and −20 kJ mol^–1^) is observed due to the relatively weak nature of dispersion-dominated interactions. For the repulsive contacts, R739×5 (Figure 8C), the highlighted ranges considering both unsigned errors and interaction energies are different, and higher relative populations for systems with errors larger than 1 and smaller than 6 kJ mol^–1^, especially around zero interaction energy, are displayed.

IHB100×10 (Figure 8H) is noticeably different because of the large number of systems with large unsigned errors and strong interactions. In this case, no remarkable population is observed for the systems with small errors, but still, it is evident that for the interactions between 0 and −40 kJ mol^–1^, errors are mainly less than 1 kJ mol^–1^. Very large unsigned errors are noted for the interactions in the range of −40 to −130 kJ mol^–1^. It should be mentioned that the actual unsigned error range for the ionic H-bonds was broader than 0–10 kJ mol^–1^, but the number of systems exceeding the upper bound (10 kJ mol^–1^) was negligible, and for the sake of consistency, they were not shown here. The same is true for some of the other figures, such as Figure 8G.

### 3.2 Variation of Dispersion and Exchange-Repulsion Terms

Among several factors affecting the shape of a potential energy surface, we investigated the nature of interactions. All multipole expansions of the polarization terms have been developed for asymptotic ranges in which coefficients can be calculated using the polarizabilities and multipole moments of isolated monomers. The asymptotic range implies that the intermonomer separations are a few times larger than the equilibrium distance. These expansions converge slowly and finally break down as the monomers become closer to each other. This can occur at distances larger than equilibrium, which are still sufficiently short to cause additional effects such as the exchange-repulsion and charge transfer. Damping functions are used to provide the correct physical picture of fittings in this range.

To gain detailed insight, we decided to investigate how dispersion decay rates are dependent on the nature of interactions in a range of distances equal to or longer than but not too far from equilibrium, which is called here the “medium-range”. For this purpose, we attempted to fit the functions of intermonomer distances to the energies obtained from the SAPT calculations. There is, of course, an important caveat to be made here that the fit coefficients presented in the following section cannot be used to recover dispersion energies in asymptotic ranges, and the use of these models is to investigate the interplay between the nature of the interactions and dispersion decay rates.

For the separations shorter than equilibrium, we analyzed the distance dependence of exchange-repulsion energy by fitting simple functions to the related SAPT data.

#### 3.2.1 Dispersion Curves

**Figure 9:**
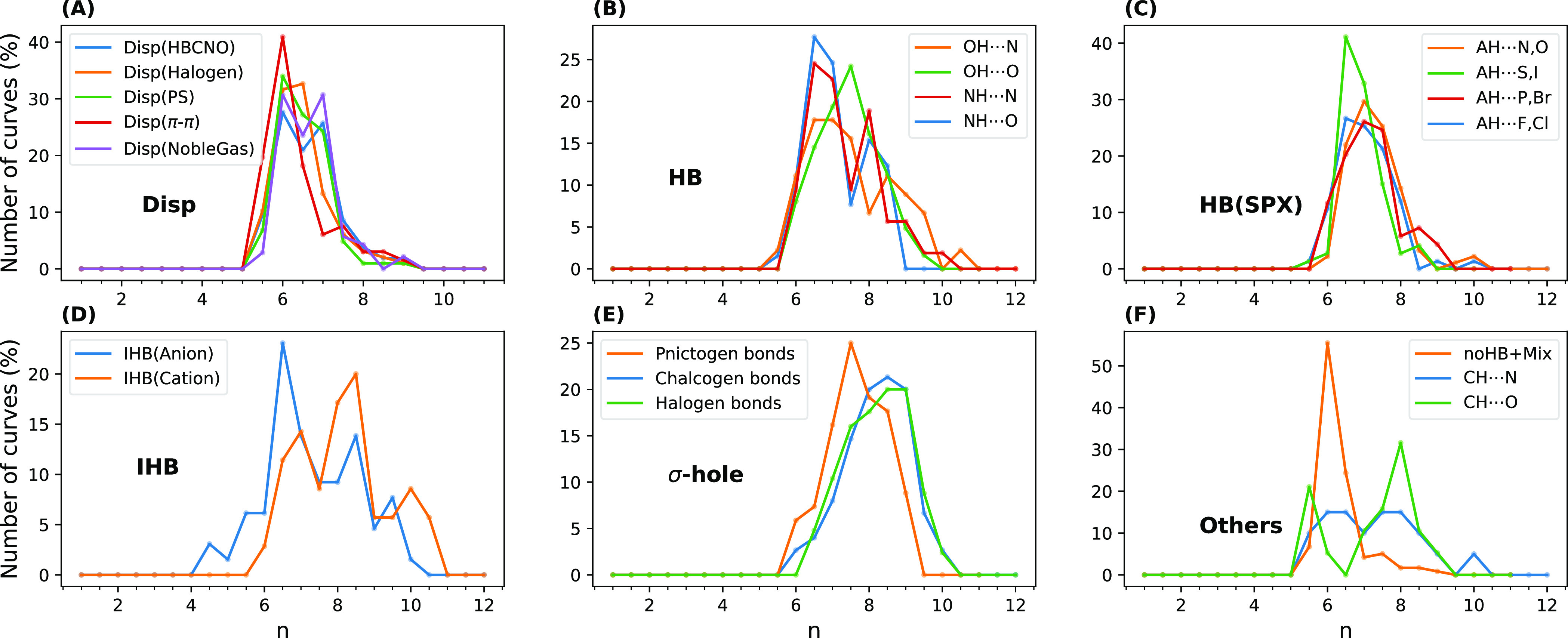
Number of dispersion energy curves (%) vs the decay rate of dispersion indicated as n in *R*^–*n*^ for each category of interaction. The SAPT calculations were performed with the PBE0AC potential.

To find the decay rates of dispersion energies, we fit a simple function with a single term like CR^–*n*^ to the SAPT dispersion energies (the details are given in the Supporting Information). Figure 9 exhibits the relative population of dispersion curves (%) in each group and subgroup of NCIs described with the best exponent (*n*) of the intermolecular COM–COM distance (*R*) in the function CR^–*n*^. In this case, the change of xc-potential causes no important differences (Figure S14). As a sufficient number of points (in our case, 5 points including the equilibrium distance) in the medium range was needed to have a reliable fit, the R739×5 and S66×8 data sets were excluded. Excellent qualities were observed for all dispersion energy fits (mostly RMSE < 0.1 kJ mol^–1^). Table S8 shows the average of the fit RMSEs for each category of interaction.

While monomer properties also affect the decay rates, the fits can be categorized in a distinguishable way only based on the type of interactions denoted in Figure 9.

As the second step, to better interpret the graphs in Figure 9, each dispersion curve was again fit to an expanded function inspired by the well-known asymptotic multipole expansion of the following form^84−87^
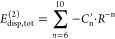
5where *C*_*n*_^′^ is defined here as the medium-ranged dispersion coefficient. It should be mentioned that *C*_*n*_^′^ is different from the well-known *C*_*n*_ coefficients related to asymptotic ranges, and eq 5 is not a functional form for the potential models. Here, we assumed that monomers are two small, rigid molecules rather than atoms or molecular sites,^39^ similar to a coarse-grained description in a molecular dynamics simulation. The relative orientation of molecules in our dimers does not change. *R* is considered to be the COM–COM distance. The total dispersion energy *E*_disp,tot_ is defined as the sum of polarization and exchange terms denoted in eq 1 as follows
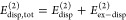
6

*E*_disp,tot_^(2)^ has the same multipole expansion as *E*_disp_^(2)^ since *E*_ex-disp_^(2)^ has no multipole expansion.^84^ Using eq 5, the weight of different terms in the decay rate of dispersion for every dimer in each group of interactions was analyzed. In Figures S12 and S13 (with a different xc-potential), the average of the contribution of the *C*_*n*_^′^ terms over the whole medium-ranged curve and in each subgroup of interaction is displayed. The range of *n* in *C*_*n*_^′^ was considered to be from 6 to 10 because Figure 9 shows that the ranges of decay rates for the systems in question are generally between these values.

Starting from the dispersion-dominated systems in Figure 9A, we observe a very sharp peak at *n* = 6 for the π–π interactions [Disp(π–π)]. It implies that the exponent 6 in the Lennard-Jones potential^91^ can be used to realistically describe the dispersion interactions in more than 40% of these systems. Figure S12 shows that, on average, more than 70% of the whole dispersion energy of the Disp(π–π) systems can be obtained from the *C*_6_^′^ term, and the contribution of *C*_10_^′^ is close to zero. In addition, *C*_9_^′^ is not an important contributor. The prominence of the *C*_6_^′^ term is reduced sequentially for Disp(Halogen), followed by Disp(PS), then Disp(NobelGas), and at last Disp(HBCNO), as a second peak appears, for example, for Disp(NobleGas) and Disp(HBCNO) at *n* = 7. However, the role of *C*_9_^′^ and *C*_10_^′^ terms is hitherto meager, and *C*_6_^′^ is the main term with a mean contribution of >50% to the whole dispersion energy considering all of the subgroups of Disp.

The Disp(π–π) subgroup is composed of those systems of D442×10 possessing at least one π-bond in each of the monomers, together with two π-stacked dimers, 1,3,5-trifluorobenzene, and hexafluorobenzene from X40×10. Thus, the systems of Disp(π–π) can also be found in other data sets and categories, such as Disp(HBCNO) and Disp(Halogen). There are some systems in Disp(π–π), which can be taken into account as π–π stacking interactions, such as toluene···benzene and 1,3,5-triazine···1,3,5-triazine dimers. For these aromatic π-stacked dimers, no contribution of the coefficients higher than *C*_6_^′^ is observed. Moreover, for the interactions such as CO_2_···cyclopentadiene and diphosphene···hydrogenazide, which are not aromatic but for which the π-bonds are still stacked, the dispersion decay rate is exactly *R*^–6^. On the other hand, higher terms become very important once there is a perpendicular or head-to-head orientation of π-systems. For example, the dispersion energy is proportional to *R*^–9^ for phosphorine···phosphorine interacting head-to-head (P···P), or *R*^–8^ in the system where CS_2_ interacts with thiophene perpendicularly.

In the case of routine H-bonds denoted with HB (Figure 9B), the importance of the *C*_6_^′^ term is low compared to the systems of the Disp group. In all of the HB subgroups, including NH···N, NH···O, OH···O, and OH···N interactions, the main contributor to the dispersion energy is the *C*_7_^′^ term. The *C*_9_^′^ and *C*_10_^′^ terms become more pronounced for the OH···N subgroup, which exhibits the highest induction and charge transfer values (Table S7). On the other hand, considering NH···O in this group as the one with the lowest mean induction and CT values, we observe the lowest contributions of the *C*_9_^′^ and *C*_10_^′^ terms, accompanied by the highest percentage for the *C*_6_^′^ and *C*_7_^′^ terms on average.

**Figure 10:**
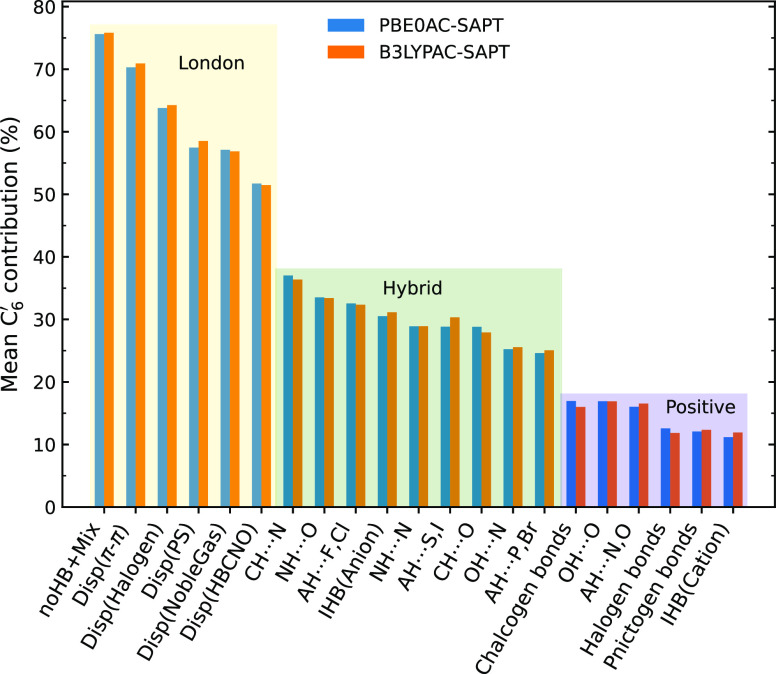
Percentage of the mean contribution of the *C*_6_^′^ term to the whole dispersion energy plotted for different interactions (the subgroups of Figure 9).

As shown in Figure 9C, the systems in the HB(SPX) group or the H-bonds containing heavier atoms, were categorized into four subgroups in terms of the amount of their induction or CT energies. Every two atoms that caused induction energies close to each other were considered the H-bond acceptors in one subgroup. Similar to the HB group, for this type of H-bond, the *C*_6_^′^ coefficient is not also the main contributor to the whole dispersion energy, and instead, *C*_7_^′^ plays the leading role. However, compared to the HB group, a very small portion of the dispersion energy can be assigned to higher terms such as *C*_9_^′^ and *C*_10_^′^ in the HB(SPX) systems. A detailed inspection (Figure 10) reveals that two extremes of charge transfer energies (Table S7), AH···N, O and AH···F, Cl interactions, exhibit the smallest and largest mean percentages for the *C*_6_^′^ term (15.8 and 32.2%) in their group, respectively. The occurrence of doubly charged anions such as carbonate and oxalate in the dimers with *n* < 6 is noteworthy (Figure 9D). Moreover, hydroxide, as a small anion with a relatively high negative charge density, is observed in the systems with this dispersion behavior. The terms between *n* = 4 and *n* = 6 are known as mixed charge-flow and dipole polarizability terms, such as, for example, charge–charge polarizabilities on one molecule and dipole–dipole polarizabilities on the other one, which lead to *R*^–5^.^84,88^ The lowest order arising from an intramolecular charge–charge polarizability leads to an *R*^–2^ contribution. Thus, an *R*^–4^ dependence can result from charge–charge polarizabilities on both monomers.

Although the conservation of charge predicts that these terms cancel each other out at large distances over all orientations, they may survive in typical configurations, especially when they are large due to the flow of extra electrons.

σ-Hole interactions show a clear tendency for higher terms (Figure 9E), and for all of them, the *C*_6_^′^ term has the smallest contribution to the dispersion energy. The *C*_8_^′^ term is the most pronounced term for all of the σ-hole subgroups. However, in the case of halogen and chalcogen bonds, which behave similarly, the contribution of *C*_9_^′^ and *C*_10_^′^ is surprisingly large. In terms of the mean contribution of every dispersion coefficient (Figure S12), they behave more or less like cationic H-bonds. Pnictogen bonds, nevertheless, are considerably different from the other two subgroups, as they exhibit the largest *C*_8_^′^ and the smallest *C*_10_^′^ contributions.

The very sharp peak at *n* = 6 in Figure 9F—which is reminiscent of Disp(π–π)—is the most interesting point in this group. As Table S7 explains, the noHB subgroup has the lowest mean induction and ind.disp^–1^ values, which are very close to those of the D442×10 data set. Consequently, the charge transfer effects are smaller compared to other subgroups in HB375×10 and insignificant. CH···O and CH···N interactions are improper or unconventional H-bonds, which are usually accompanied by an IR blue-shift in the proton donor moiety and different charge transfer features.^89,90^

Figure 10 displays the contribution of the *C*_6_^′^ term to the dispersion energy in the medium range for all of the interaction subgroups, sorted from the highest to the lowest. The very weak interactions (noHB+Mix) and positively charged H-bonds are located in the first and last positions, respectively. Although the effects of monomer properties come into play when discussing the decay rate of dispersion energy, our investigation shows that particular NCIs have a distinguishable distance-dependent dispersion energy. As shown in Figure 10, however, NCIs can fall into three categories in terms of *R*^–6^ dependence. In this respect, the first category covers all of the dispersion-dominated interactions and very weak H-bonds, with the *C*_6_^′^ contribution ranging from 50 to 75%. There is a good probability in this scope to find a dimer whose dispersion can be safely described with the well-known formula, *R*^–6^. The second scope allows for different types of H-bonds, including improper, anionic, and ordinary H-bonds. For this range, the contribution of the *C*_6_^′^ term is between 20 and 40%. To treat the dispersion energy of these systems, a combination of different coefficients, including *C*_6_^′^, is necessary. The third scope belongs to the systems whose dispersion energy is not significantly dependent on the *C*_6_^′^ term. In return, higher terms such as *C*_8_^′^ and even *C*_10_^′^ are of great importance. σ-hole interactions with the anisotropic positive charges are all members of this group, and cationic H-bonds have even smaller *R*^–6^ dependences due to their high positive electrostatic potentials. The *C*_6_^′^ contribution for these systems is less than 20%. AH···N, O and OH···O also fall into this scope, probably because of their high induction values and some effects related to their monomer properties. However, these two subgroups are different from the other subgroups in terms of their higher *C*_7_^′^ contributions. We called the NCIs falling within these three scopes: London, hybrid, and positive interactions.

#### 3.2.2 Exchange-Repulsion Curves

For many years, simple models such as *A*/*R*^12^ in the Lennard-Jones equation^91^ and *A* exp(−*BR*) in the Born–Mayer equation have been very popular for the description of short-range forces.^92^*A*/*R*^12^ is an example of the repulsion model in the Mie potential, *A*/*R*^*n*^.^93^ In this work, we attempted to fit these models in the form of molecule–molecule interactions to the first-order exchange-repulsion terms obtained from our SAPT calculations (*E*_ex_^(1)^).

**Figure 11:**
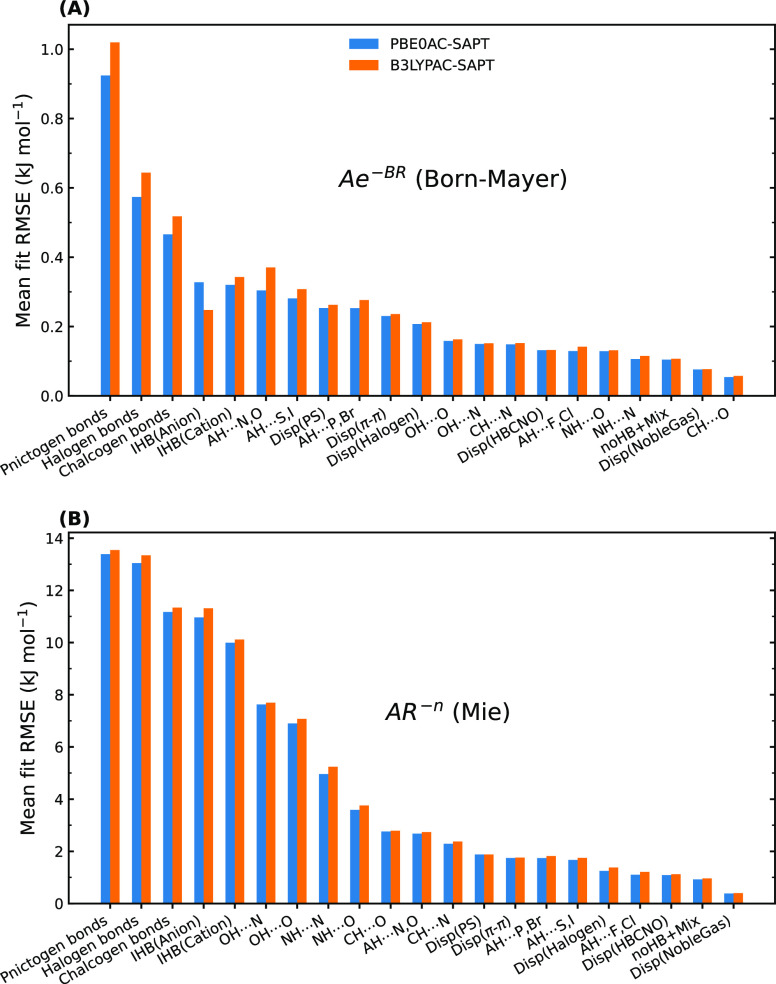
Comparison of the performance of the Born–Mayer (*Ae*^–*BR*^) and Mie (*AR*^–*n*^) models in describing intermolecular exchange-repulsion by benchmarking against the SAPT data considering both xc-potentials.

Figure 11A reveals the excellent performance of the Born–Mayer model with two fitting parameters, *A* and *B*, in describing the SAPT exchange-repulsion energies for all the interactions with the mean fit RMSE less than 1.0 kJ mol^–1^. *B* turned out to be a value between 2.23 and 5.04 Å^–1^, with the average equal to 3.1 Å^–1^. The best fits are associated with CH···O, Disp(NobleGas), and noHB+mix subgroups, while σ-hole interactions exhibit the highest RMSE values.

The Mie repulsion model (Figure 11B), however, may not be considered a model of interest for most of the interactions, especially σ-hole interactions and charged H-bonds. This model can be suitable only for a few subgroups such as Disp(NobleGas), noHB+Mix, Disp(HBCNO), and AH···F, Cl. Figures S16 and S15 show how the exponent of *R* varies in terms of different types of interaction, keeping in mind that the fit RMSE for many of them is relatively large. For the interactions mentioned above that have the best accuracy, the decay rate varies mostly from *R*^–8^ to *R*^–12^. For all other systems, depending on the type of interaction, orientations, and monomer properties, the exponent can vary from −7 to −15. Nevertheless, finding a good fit for the special case of *R*^–12^ is very difficult, as Figure S17 evidently displays huge errors for the majority of subgroups. According to these results, the Lennard-Jones repulsion model may be suitable only for some of the rare gas dimers.

Although most force fields fitted to ab initio data use functional forms much more complicated than Lennard-Jones or Born–Mayer, for coarse-grained models and very large simulations where a whole molecule has certain coefficients assigned, simple models are preferred.

### 3.3 Data Reduction

A data set representing all noncovalent interactions with the highest coverage and lowest redundancy is of great importance to test newly developed methods. Here, we utilized the relative entropy or Kullback–Leibler divergence between the distributions of two data sets in the feature domain to determine which set could better represent the reference data set.^94^ In total, 119 features were chosen to cluster the data. The whole procedure is described in the Supporting Information.

**Figure 12:**
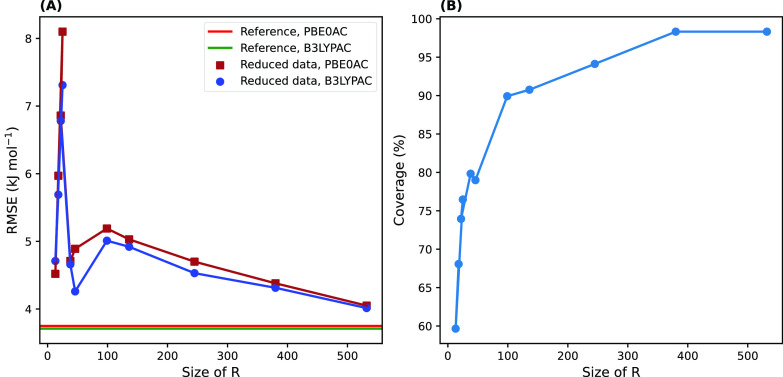
(A) Total RMSE values of DFT-SAPT results with two employed xc-potentials for each representative data set compared to those of the reference set with a size of 1507 curves (horizontal red and green lines). The horizontal axis shows the number of dissociation curves in each data set. (B) The change in coverage percentage was proportional to the size of the representative data sets.

As shown in Figure S1, the best coverage (98.32%) belongs to a data set with 532 potential curves out of 1507. It means that with a 65% reduction in the size of the original total data set (collection of all the systems with 10 point dissociation curves), only less than 2% coverage loss is incurred. However, smaller data sets that can be computed rather quickly are of much interest for testing a methodology in quantum chemistry. We expect that the measured accuracy with application of a method of choice on a small representative set will be comparable to the accuracy associated with a comprehensive and large reference. In Figure 12A, total RMSE values related to each reduced data set were compared to the reference values. For the data sets larger than 100, the RMSE values approach the reference values in a linear manner for both xc-potentials. Figure 12B also shows how the coverage percentage increases with an increase in the size of data sets. Given these relations, we decided on the data set with the size 245 as an optimum choice, which is sufficiently reduced, whereas the coverage percentage is still desirable (94.1%), and the difference between its total RMSE values and the reference ones is less than 1 kJ mol^–1^ (0.82 and 0.95 kJ mol^–1^ for the B3LYPAC and PBE0AC potentials, respectively).

## 4 Conclusions

We created a comprehensive set of high-quality DFT-SAPT data for nearly all types of noncovalent interactions and their dissociation curves. Among the calculated systems are σ-hole interactions with a large amount of charge transfer, singly and doubly charged H-bonds, and relatively large molecules with heavy atoms, which cause many computational challenges for DFT-SAPT.

Both methodological aspects and individual contributions of the interactions were investigated, and all DFT-SAPT total interaction energies were benchmarked against the best available CCSD(T)/CBS values. From a methodological point of view, the effect of the underlying xc-potential was assessed using PBE0 and B3LYP in their asymptotically corrected (AC) form. Our studies showed that for nonequilibrium distances, B3LYPAC, on average, outperforms PBE0AC. B3LYPAC is also the method of choice for dispersion-dominated systems, repulsive contacts, and the majority of neutral H-bonds. However, PBE0AC is superior to B3LYPAC for ionic H-bonds and σ-hole interactions, especially for distances close to equilibrium. Thus, the DFT-SAPT results are rather dependent on the type of exchange–correlation potential.

In spite of its exact form in the infinite expansion of the exchange term, the use of *S*^∞^ in induction and dispersion does not yield more accurate results than does the *S*^2^ approximation. *S*^∞^ leads to less accurate total interaction energies for many H-bonded systems, especially for ionic H-bonds and when B3LYPAC is used. This can be attributed to the error cancellation between the *S*^2^ approximation and the used xc-potential.

Neutral noncovalent interactions containing only the first-row nonmetals and H are the ideal systems for DFT-SAPT. On the other hand, halogen bonds, H-bonds containing heavier atoms, ionic H-bonds, and repulsive contacts should be dealt with carefully. Still, for pnicogen and chalcogen bonds, accurate results can be obtained using regularized SAPT.

When H-bonded monomers containing heavier atoms become very close to each other, the covalent nature of the interaction may increase significantly, accompanied by a proton transfer, and this causes the lower-order SAPT methods with only the δHF correction to highly deviate from the benchmark results. In these cases, the problem can be cured by using higher-order SAPTs such as SAPT2+(3) or SAPT2+3 plus a δMP2 term.

Dimers exhibit particular dispersion decay rates in terms of the nature of their interactions over medium-range intermonomer distances. In this respect, they fall into three large categories that we called London, hybrid, and positive interactions. London interactions consist of very weak H-bonds, π–π interactions, dimers containing noble gases, etc. The dispersion energy of these complexes mainly decays as *R*^–6^. As a member of the hybrid category, nearly all H-bonds turn out to have significant portions of *R*^–7^, *R*^–8^, and sometimes higher terms. The last category includes interactions with positive or partial charges responsible for most of the attractive force, thus decaying faster with decreasing intermonomer distances. σ-hole interactions and cationic H-bonds exhibit dispersion decay rates with small portions of the *R*^–6^ term.

When considering the contribution of the *R*^–6^ term to the dispersion energy in the medium range and the fit of the Born–Mayer or Mie models to exchange-repulsion energies in the short range, the importance of charge-transfer effects was observed in many cases.

Finally, from a data reduction perspective, a newly extracted data set called “noncovalent interactions for DFT-SAPT” (NDS245×10) can be recommended for testing or validation processes. As a representative of all types of intermolecular interactions, the NDS245×10 data set is a reliable platform, especially when different categories of H-bonds must be considered.

## Data Availability

All the calculated energy values (kJ mol^–1^) along with the COM–COM distances (Å), and the data of fitting procedures are available as the Supporting Information. A part of the data is also available in the GitHub repository (https://github.com/Honza-R/NCIAtlas) linked to the web page of the NCIs Atlas project (http://www.nciatlas.org/).

## Supporting Information Available

The geometries of the dimers as the representative data set (NDS245×10) in the xyz format (ZIP)
The fit data for the exchange-repulsion curves (ZIP)
The fit data for the dispersion curves (ZIP)
The COM-COM distances, DFT-SAPT energies, and reference total energies (ZIP)
Additional materials and notes on extracting a representative data set, relative errors and ranking, fitting procedure, π-π interactions, distribution in error ranges, errors vs. charge transfer, problematic systems, average of some energy terms, intermolecular distances, fit data, and Molpro vs. Psi4 (PDF)
