# Supplementary material for: Benchmarking Swaths of Intermolecular Interaction Components with Symmetry-Adapted Perturbation Theory
Source: J Chem Theory Comput. 2023 Dec 20;20(1):30–48. doi: 10.1021/acs.jctc.3c00801 (PMC10782453; doi:10.1021/acs.jctc.3c00801)
Supplement: Supplementary file 5 — ct3c00801_si_005.pdf [file ct3c00801_si_005.pdf]

Supporting Information for "Benchmarking Swaths of Intermolecular Interaction Components with Symmetry-Adapted Perturbation Theory"

Ehsan Masumian* and A. Daniel Boese*

Physical and Theoretical Chemistry, Department of Chemistry, University of Graz, 8010 Graz, Austria

E-mail: ehsanmasumian@yahoo.com; adrian_daniel.boese@uni-graz.at

This paper also includes the following data as zip files:

- The fit data for the dispersion curves
- The fit data for the exchange-repulsion curves
- The geometries of the systems in the representative data set
- The energies and intermolecular distances

Contents

1 Extracting a representative data set **3**

1.1 Greedy algorithm 3

1.2 Chosen features 5

1.3 Validation 7

2 Relative errors and ranking **8**

3 Fitting procedure **9**

4 Additional figures and tables **11**

4.1 π-π interactions 11

4.2 Distribution in error ranges 11

4.3 Errors *vs.* charge transfer 16

4.4 Problematic systems 17

4.5 Average of some energy terms 20

4.6 Intermolecular distances 21

4.7 Fit data 25

4.8 Molpro *vs.* Psi4 30

5 References **30**

1 Extracting a representative data set

1.1 Greedy algorithm

Two main factors were considered to be optimum when searching for a good representative data set: high data coverage and low redundancy [1]. The core quantity used to estimate these criteria is the relative entropy or Kullback–Leibler divergence (D_{kl}) calculated as follows:

$$D_{kl}\left[E_i \| E_j\right] = \sum_{f \in F} P\left(f|E_i\right) \log \frac{P\left(f|E_i\right)}{P\left(f|E_j\right)} \tag{S1}$$

where, E_i and E_j are two element sets belonging to E (the original data set), and $P(f|E_i)$ is the conditional probability obtained from the following expression:

$$P\left(f|E_i\right) = \frac{1}{|E_i|} \sum_{e \in E_i} P\left(f|e\right) \tag{S2}$$

where $P\left(f|e\right)$ is the conditional probability of the element e possessing the feature f, which is equal to $1/|F_e|$ with $|F_e|$ being the number of items in the feature set associated with the element e. $|E_i|$ is the number of items in the element set i.

The mutual information of X and Y (discrete random variables) is given as

$$I(X,Y) = H(X) - H(X|Y) \tag{S3}$$

where H(X) is the Shannon entropy of X, and $H(X|Y)$ is the entropy of Y conditioned on X. The description of these quantities can be found in every related text in the literature. $H(X)$ is omitted if we want to obtain the difference between two $I(X,Y)$ values, which are only different in Y:

$$\Delta I(Y_2, Y_1) = H(X|Y_1) - H(X|Y_2) \tag{S4}$$

To measure how better one representative data set is than the other one in capturing information from the original data set, we can compare their related mutual information. If we consider two representative sets which are different only in one element, $R_2 = R_1 \cup r_{n+1}$, then the difference between their mutual information can be written as follows:

$$\Delta I(Y_2, Y_1) = \frac{1}{|E|} \sum_{f \in F} \sum_{e \in E_{2\theta}} P(f|e) \log \frac{|E_{1\theta}| \sum_{e \in E_{2\theta}} P(f|e)}{|E_{2\theta}| \sum_{e \in E_{1\theta}} P(f|e)} + \frac{1}{|E|} \sum_{f \in F} \sum_{e \in L(r_{n+1})} P(f|e) \log \frac{|E_{1\theta}| \sum_{e \in L(r_{n+1})} P(f|e)}{|L(r_{n+1})| \sum_{e \in E_{1\theta}} P(f|e)} \tag{S5}$$

which is obtained from combining equations S1, S2, and S4. $E_{1\theta}$ and $E_{2\theta}$ are two sets covering the elements which are related to none of elements in R_1 and R_2 sets, respectively. Y_2 and Y_1 are two random variables defined over the representative sets of R_2 and R_1 respectively. $L(r_{n+1})$ is a set of elements related to the representative element r_{n+1}. To determine the related set for a representative r, $D_{KL}(r||e_i)$ is to be calculated between r and every other element, e_i, which is not in the representative set or not related to any other representatives, and then the minimum ($\min D_{KL}$) is chosen. Those elements whose D_{KL} is equal to or less than $\min D_{KL} \times t_{max}$ are taken into account as the related set for r. t_{max} is a parameter with the value ≥ 1 to control the range of selection.

Another measure that must be addressed for selecting a decent representative set is redundancy. To maximize the difference between a new r and the previously chosen representatives the relative entropy between them must be the highest. In this regard, the minimum of all the calculated $D_{KL}(r_{new}||r)$ values is considered to be maximized. Taking the above-mentioned point an objective function can be defined as follows:

$$f(r_{new}, R) = \Delta I(Y_{new}, Y) + \min_{r \in R}(D_{KL}(r_{new}||r)) \tag{S6}$$

The higher the value of this function is, the less redundancy and the more coverage are obtained by selecting a new r. Therefore, the main task to find the most appropriate rep-

resentative is to maximize this function. However, there might be many items with the maximum objective function, of which we decided to select those with the highest number of associated features in order to control the number of added representatives while covering the largest number of features at each time.

All the zero probabilities were replaced with 1.0e-10 to avoid undefined terms. In addition, because when the value of a relative entropy is zero the use of parameter t_{max} is in fact pointless, all the relative entropies were increased by 1.0.

1.2 Chosen features

We attempted to consider an ample number of diverse features to describe the systems. Basically, they were categorized into 7 classes: type of interaction, type of element, number of atoms (size), interaction energy, accuracy of SAPT, ind.disp^{-1} ratio, and original data sets, which in total contain 119 features as shown in Table S1. All the features in this study are basically unweighted. However, we decided to emphasize on the class *type of interaction* to avoid not representing a system with another system possessing a different type of interaction. For this purpose, in addition to the features that show the interaction form very specifically, a general description of them was also given as a distinct feature. For example, for an ethyne-acetate dimer, the interaction type is described using three features **HB**, *IHB*, HB_anion. Moreover, the name of original data set as a feature weighs the interaction type, which is in this case IHB100×10.

According to Table S1 the presence of any elements except for H and C was considered as another feature. Three ranges were chosen for the size of dimers, and 17 ranges were considered to categorize interaction energies denoted with the absolute values for the equilibrium structures. Each feature related to *accuracy of SAPT* consists of the name of underlying xc-potential, the range of distances (all the dissociation curve points (all) or only minimum point (min)), and the level of accuracy as described in Table S4. ind.disp^{-1} ratios were grouped into 14 ranges, and calculated with the PBE0AC potential. The data sets S66x8

and R739x5 were excluded since their dissociation points were not consistent with other 10 point curves.

Table S1: The features chosen to extract the representative data set using the Greedy algorithm classified into 7 groups. The features in bold include a wide range of other features coming after them until the next bold feature. The features in italic also contain a deeper sub-group of the follow-up features until the next bold or italic feature. *all* and *min* denote all distances and minimum point, respectively. Δ means the difference between PBE0AC-SAPT and B3LYPAC-SAPT results.

Class	Features
Type of interaction	**HB**, CHN, CHO, NHN, NHO, OHN, OHO, XHBr, XHCl, XHF, XHI, XHN, XHO, XHO, XHP, XHS, *IHB*, HB_anion, HB_cation, *noHB*, **Disp**, Disp_halogen, Disp_HBCNO, Disp_noblegas, Disp_$\pi\pi$, Disp_PS, **σ-hole**, *halogen_bond*, σ-hole_Cl, σ-hole_Br, σ-hole_I, *chalcogen_bond*, σ-hole_S, σ-hole_Se, *pnictogen_bond*, σ-hole_As, σ-hole_P, **Mix**
Type of element	He, Ne, Ar, Kr, Xe, F, Cl, Br, I, O, S, Se, N, P, As, B
Number of atoms	small $<$ 15, 15 $\leq$ medium $<$ 25, 25 $\leq$ large
Interaction energy	0-10, 10-20, 20-30, 30-40, 40-50, 50-60, 60-70, 70-80, 80-90, 90-100, 100-110, 110-120, 120-130, 130-140, 150-160, 160-170, 170-180
Accuracy of SAPT	PBE0_all_excellent, PBE0_all_verygood, PBE0_all_good, PBE0_all_fair, PBE0_all_poor, PBE0_min_excellent, PBE0_min_verygood, PBE0_min_good, PBE0_min_fair, PBE0_min_poor, B3LYP_all_excellent, B3LYP_all_verygood, B3LYP_all_good, B3LYP_all_fair, B3LYP_all_poor, B3LYP_min_excellent, B3LYP_min_verygood, B3LYP_min_good, B3LYP_min_fair, B3LYP_min_poor, Δ_all_excellent, Δ_all_verygood, Δ_all_good, Δ_all_fair, Δ_all_poor
ind.disp^{-1} ratio	0.0-0.25, 0.25-0.5, 0.5-0.75, 0.75-1.0, 1.0-1.25, 1.25-1.5, 1.5-1.75, 1.75-2.0, 2.0-2.25, 2.25-2.5, 2.5-2.75, 2.75-3.0, 3.0-3.25, 3.25-4.0
Original data set	IHB100×10, HB375×10, HB300SPX×10, SH250×10, D442×10, X40×10

1.3 Validation

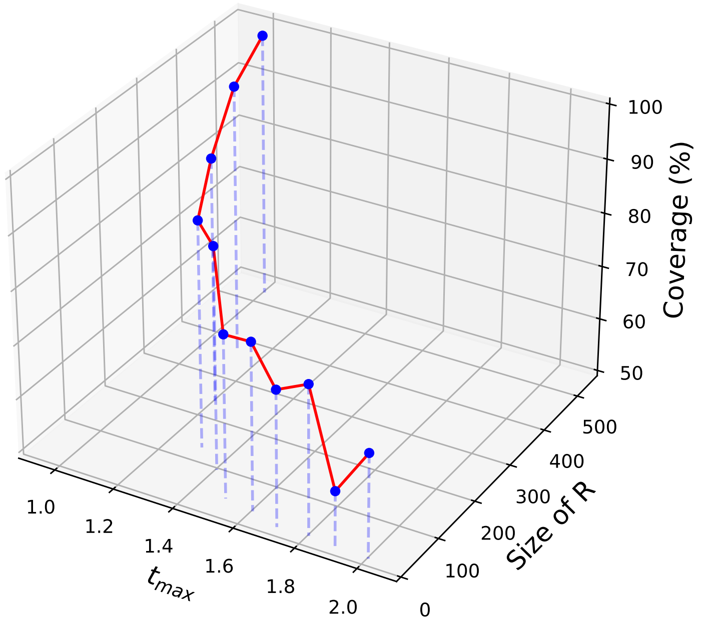

Figure S1: The correlation between three parameters: 1. The coverage of features by the generated representative data set (R), 2. The number of individual dimers (potential curves) in R, and 3. The criterion used to control the range in which representative elements were picked up, t_{max}.

The original set of data selected as the reference is composed of the collection of 6 data sets namely IHB100×10, HB375×10, HB300SPX×10, SH250×10, D442×10, and X40×10. This total set amounts to 1507 potential curves each at 10 different distances. It is expected that this collection covers nearly all types of non-covalent bonds. 11 reduced data sets with different sizes were generated by choosing a range of t_{max} values from 2.0 to 1.0.

In order to validate the performance of the generated representative data sets we established two criteria namely coverage percentage and the closeness of total RMSE values to those of the reference set. The coverage was introduced as the number of features appeared in the representative data set divided by the total number of features. In Fig. S1, the variation of coverage percentage with t_{max} and the size of representative data set is visualized. For the higher t_{max} values some fluctuations are observed, but in general the coverage increases with

decreasing the t_{max} values and increasing the size of representative data set. The RMSE values were calculated for each generated representative data set with two xc-potentials, and are shown in the main manuscript.

2 Relative errors and ranking

Since there is a wide range of interaction types with very different magnitudes of energy, the analysis of relative errors in addition to unsigned errors is required to reasonably assess the performance of the given methods when comparing different non-covalent interactions. As the routine relative error formula gives rise to a singularity when the reference interaction energy crosses zero, Patkowski et al. proposed a mean capped unsigned relative error (MCURE) to circumvent this issue [2,3]:

$$\begin{aligned} MCURE &= 100\% \cdot \frac{1}{N}\sum_{i=1}^{N}\left|\frac{E_i^{int} - E_i^{int,ref}}{E_i^{weight}}\right| \\ E_i^{weight} &= \max\left[\left|E_i^{int,ref}\right|, \frac{\xi\left|E_i^{int,ref-eq}\right|}{z_i^3}\right] \end{aligned} \tag{S7}$$

where $z_i = R_i/R_{eq,i}$ is the ratio of the intermonomer-separation R_i of the given dimer to its corresponding equilibrium distance, E_i^{int} is the interaction energy calculated with the method of interest (here DFT-SAPT), $E_i^{int,ref}$ is the reference interaction energy, and $E_i^{int,ref-eq}$ is the difference between the equilibrium interaction energy and that of the given distance. ξ is a dimensionless parameter to mitigate the aggressiveness of the capping and was set to 0.2 according to ref [2,3]. We utilized this formula to calculate the relative errors for all the data sets used in this work. For the R739×5 data set we used the interaction energy of the dimer with the distance factor 1.00 in place of the equilibrium energy. However, because MCUREs for repulsive contact without any distinct equilibrium distance could be disputable especially when their total interaction energies are very close to zero, we reported the total MCURE (for All) once with and once without the inclusion of R739×5.

We chose a scheme based on the MCURE and RMSE values considering the two xc-potentials for ranking different types of non-covalent bonds in order of the success of DFT-SAPT in predicting their interaction energy. In this scheme, each interaction type was placed 4 times in rank order from 0 to 34 based on the errors of the related methods: RMSE(PBE0AC-SAPT), RMSE(B3LYPAC-SAPT), MCURE(PBE0AC-SAPT), and MCURE(B3LYPAC-SAPT). These 4 numbers were equally weighted and added together to provide each group of interaction with one single number. The groups were then ordered according to these numbers and normalized by dividing by 11, which is the lowest number belonging to Mix with the best rank.

To investigate the relative charge transfers *vs.* the relative errors we adopted a similar pathway to circumvent the related singularities. The charge transfer percentages were thus calculated by replacing the $E_i^{int} - E_i^{int,ref}$ expression with the charge transfer energy (E_{CT}) in Eq. S7. The resulting plot is shown in Fig. S5B. The induction percentages were also calculated in this way (Table S7).

3 Fitting procedure

The *curve_fit()* function from the SciPy library was used to fit the curves to the data points obtained from the SAPT calculations. This function applies non-linear least squares to fit the data to a function. No initial guess and no y data's level of uncertainty were given. The *maxfev* parameter was set to 10^6, which indicates the maximum number of function calls. Only those data sets with 10 dissociation points were considered for this purpose.

In the case of dispersion fit, all the energy points with the COM-COM distances longer than and including the equilibrium distance amounting to 5 points (the related distance scaling factors: 1.00, 1.05, 1.10, 1.25, 1.50, 2.00) were selected. First we tried to fit a simple function containing only two fit parameters ($\mathrm{CR^{-n}}$) and another time to fit a function of 5 terms with the exponents from -6 to -10 ($-\sum_{\mathrm{n}=6}^{10} \mathrm{C'_n R^{-n}}$) including 5 fit parameters ($\mathrm{C'_n}$ coefficients).

In the first case, each curve was fitted 38 times and each time with a different exponent varying between 2.0 and 20.0 (2.00, 2.50, 3.00, 3.50, ..., 20.0), of which the fit with the minimum RMSE was chosen and the fit parameters were determined based on the related function. The average of RMSEs for all the categories is shown in Table S8. No upper and lower bounds were imposed on the function during the fitting process. For the case of expanded terms, the lower bound was set to zero and the upper bound was set to infinity for only considering the positive values. Only one time the fitting function was used for each curve. The RMSEs and all the fit parameters are available in the separate data files. The coefficients of the expansion were chosen to be positive, and thus the negative sign in the related equation is necessary.

In the case of exchange-repulsion fit, all the energy points with the COM-COM distances shorter than the equilibrium distance together with the equilibrium energy were chosen which are 5 points indicated with 0.80, 0.85, 0.90, and 0.95 and 1.00 distance scaling factors. Three function forms were used to fit the exchange curves including AR^{-n}, AR^{-12}, and $\mathrm{Ae}^{-\mathrm{BR}}$ representing Mie (two fit parameters), lennard-Jones (one fit parameter), and Born-Mayer (two fit parameter) potentials.

4 Additional figures and tables

4.1 π-π interactions

Table S2: The dimers chosen as systems with π-π interactions.

Category	Dimers
Disp(π-π)	**D442×10**: 1.06.37, 2.13.20, 2.08.11, 2.06.11, 1.07.62, 1.07.72, 1.06.01, 1.14.06, 2.09.06, 1.10.07, 2.03.22, 3.05.13, 2.03.45, 2.09.12, 1.07.56, 1.11.12, 1.06.64, 1.07.74, 2.04.62, 1.12.09, 2.08.06, 2.07.04, 2.12.21, 2.05.06, 2.12.15, 2.06.17, 1.07.45, 1.06.17, 2.03.31, 2.04.71, 1.06.63, 2.04.02, 2.04.52, 1.10.03, 1.07.70, 1.07.55, 2.03.57, 2.13.11, 1.07.06, 2.04.38, 1.09.03, 2.06.15, 2.03.34, 2.05.13, 1.09.16, 1.12.11, 2.05.11, 1.10.01, 2.12.11, 1.07.83, 3.10.01, 1.10.05, 2.05.20, 2.10.01, 1.06.22, 1.09.35, 2.05.18, 2.05.19, 1.11.09, 2.03.55, 1.08.39, 2.11.01, 1.10.10, 2.06.22; **S66×8**: 24, 25, 26, 27, 28, 29, 30, 31, 32, 33; **X40×10**: 11, 12

4.2 Distribution in error ranges

Table S3: The number of positive and negative values (error differences) in Fig. S3 for each subplot ((**A**), (**B**), (**C**), and (**D**)). Here, total energies are without the δHF term.

	**A**			**B**			**C**		**D**	
category	(+)	(-)	(0)	(+)	(-)	(0)	(+)	(-)	(+)	(-)
IHB	47	953	0	43	957	0	124	876	127	873
HB	278	2136	0	240	2172	2	261	2153	260	2154
Disp	367	1313	0	303	1366	11	366	1314	360	1320
Others	465	759	10	269	954	11	409	825	247	987
All	1157	5161	10	855	5449	24	1160	5168	994	5334

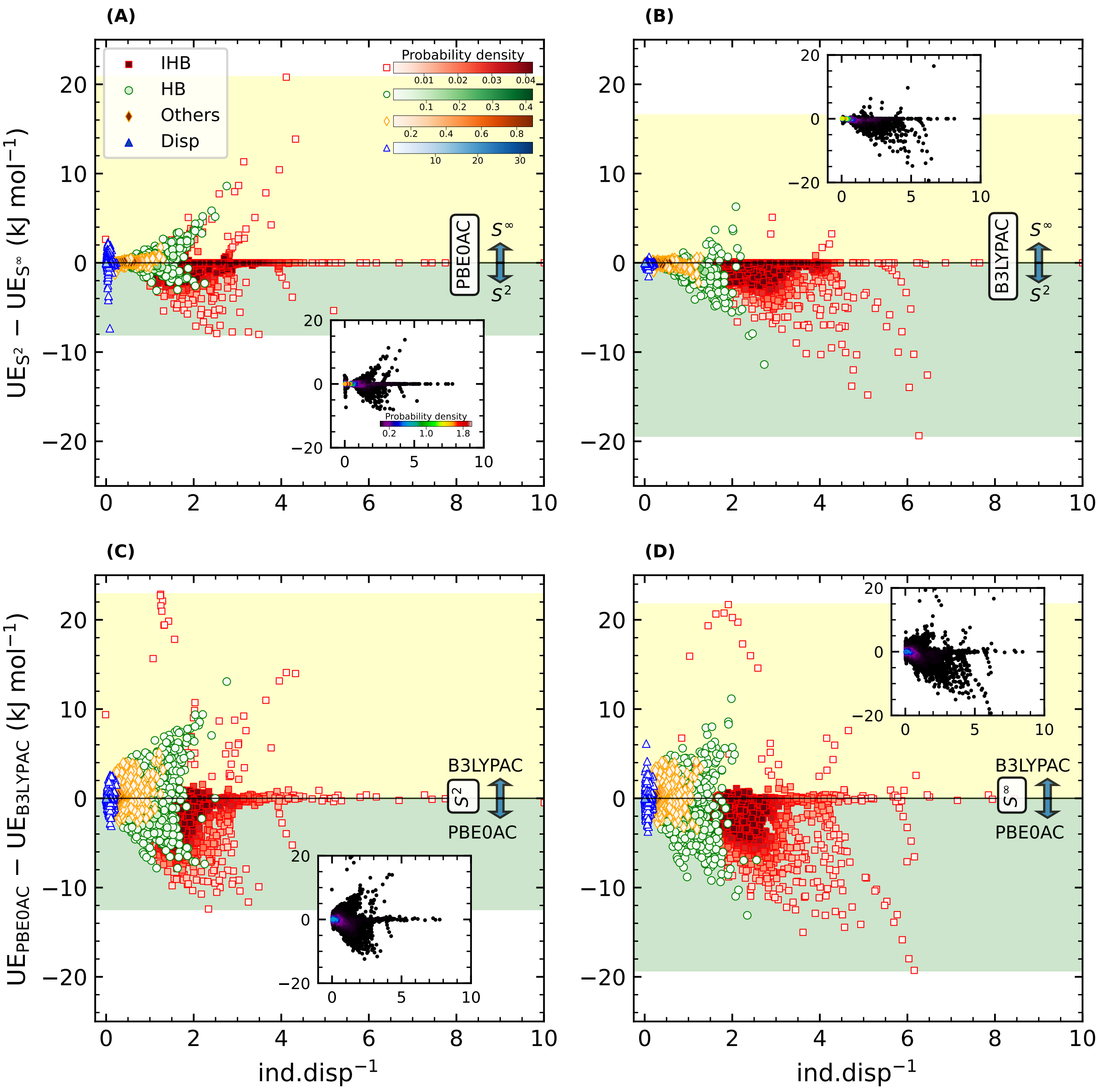

Figure S2: Fig. 1 in the main text including color bars and insets demonstrating the whole density variations. The densities were analyzed using the KDE approach once for each category and once for all the data points.

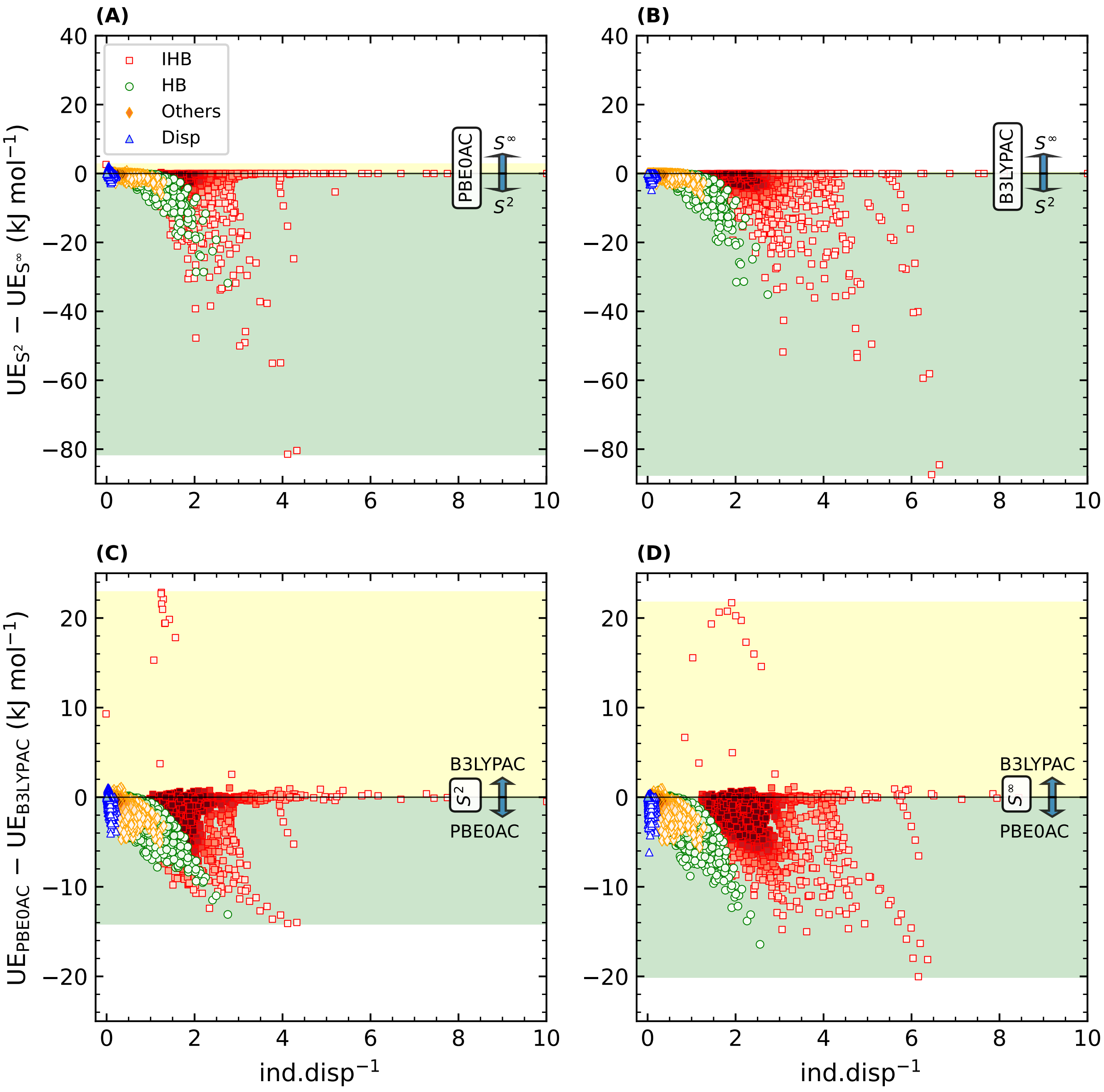

Figure S3: The representation of Fig. 1 (main text) based on the interaction energies without the inclusion of δHF.

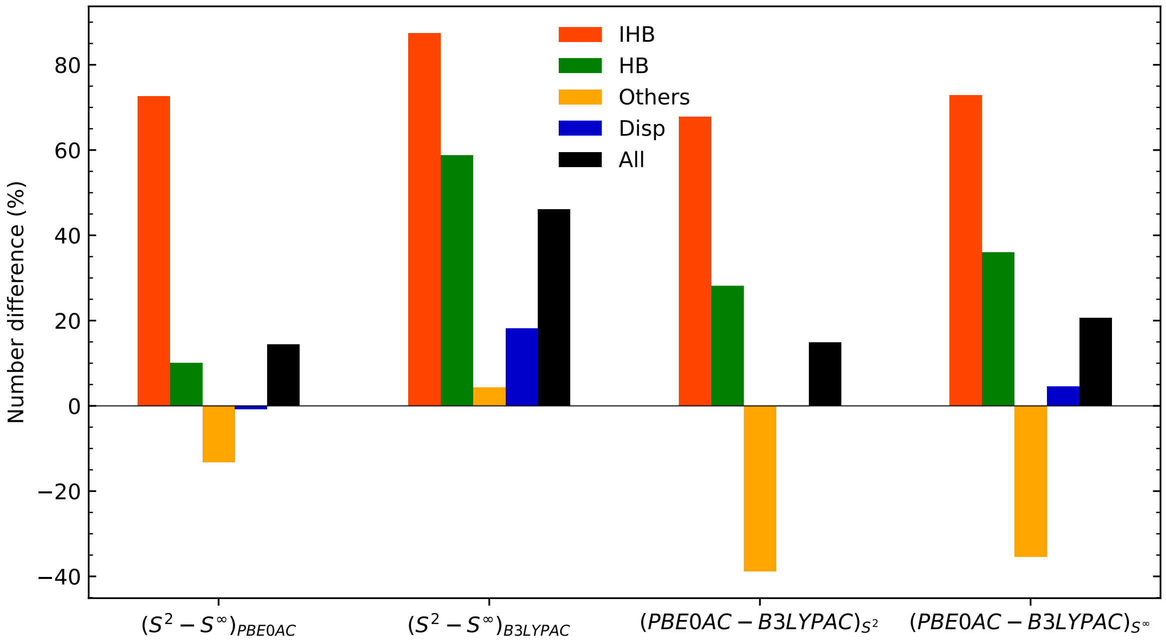

Figure S4: The calculated percentage differences as Number difference (%) = ((number of negative values - number of positive values)/ number of all values) $\times$ 100 using the data of Table 3 in the main text.

Table S4: The number of dimers associated with each RMSE range of the results obtained from the SAPT calculations with PBE0AC(S^2); excellent ≤ 1, $1 <$ very good ≤ 2 , $2 <$ good ≤ 4, $4 <$ fair ≤ 7, poor > 7 kJ mol^{-1}. The data in parentheses are for B3LYPAC(S^2).

Data set	excellent	very good	good	fair	poor
IHB100×10	2(1)	11(4)	32(18)	34(33)	21(44)
S66×8	55(36)	8(24)	3(2)	0(4)	0(0)
HB375×10	184(198)	116(97)	60(65)	15(15)	0(0)
HB300SPX×10	44(88)	69(81)	97(78)	59(35)	31(18)
R739×5	243(275)	198(223)	272(221)	20(17)	6(3)
SH250×10	31(33)	54(57)	82(79)	57(50)	26(31)
D442×10	200(262)	122(84)	85(73)	28(16)	7(7)
X40×10	12(13)	9(10)	12(12)	5(5)	2(0)
All	771(906)	587(580)	643(548)	218(175)	93(103)

Table S5: MCURE and RMSE values of the results obtained from the SAPT calculations with PBE0AC(S^2) and B3LYPAC(S^2) in parentheses.

Data set	MCURE (%)	RMSE (kJ mol^{-1})
IHB100×10	7.33(10.65)	6.25(7.87)
HB375×10	8.29(6.71)	1.74(1.74)
HB300SPX×10	25.69(19.6)	5.07(4.15)
R739×5	47.29(44.69)	2.22(1.99)
SH250×10	18.69(19.21)	4.77(5.00)
D442×10	22.2(19.67)	2.32(1.98)
S66×8	4.52(6.18)	0.92(1.83)
X40×10	17.73(16.7)	3.08(2.55)
All	23.04(20.97)	3.46(3.41)
All − R739×5	17.3(15.36)	3.69(3.66)

4.3 Errors *vs.* charge transfer

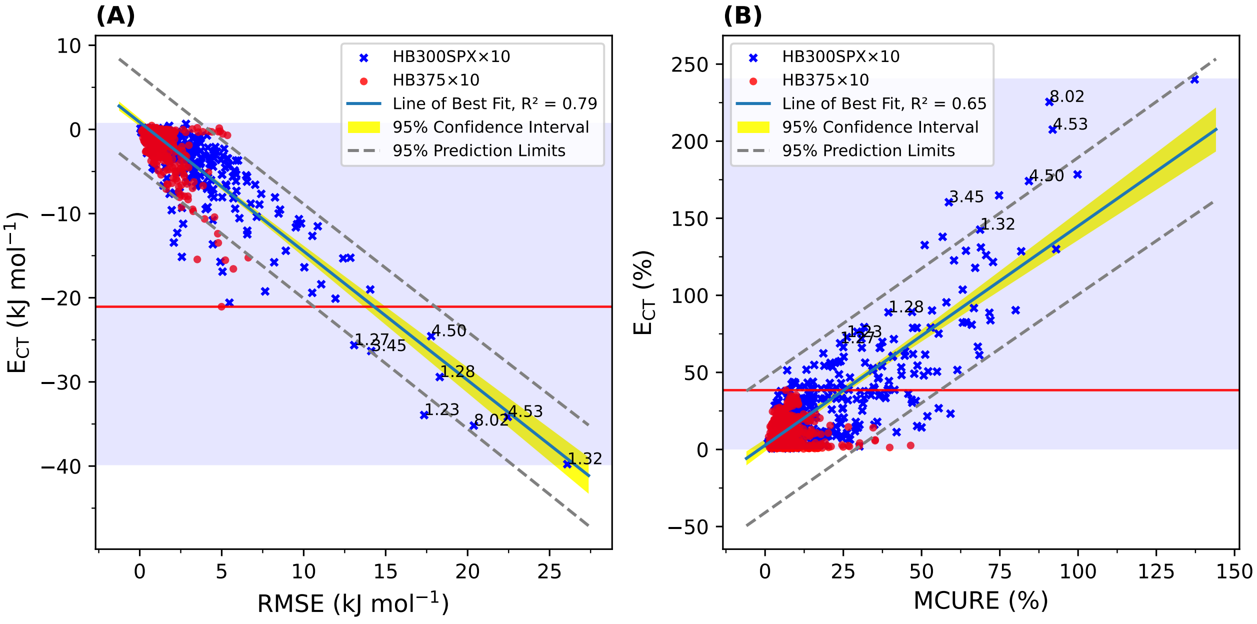

Figure S5: (**A**): The average of charge transfer energies *vs.* the root mean squared error (RMSE) over each dimer potential curve. (**B**): The capped mean charge transfer percentage, E_{CT} (%), *vs.* The MCURE (%) for each dimer curve. E_{CT} (%) was calculated based on the same formula as used for calculating MCUREs. The dimers in HB300SPX×10 having charge transfer energies out of the range of the HB375×10 charge transfer values were annotated with their related numbers in (**A**) and then they were also indicated in (**B**). For all the SAPT calculations here the PBE0AC potential was used with the aug-cc-pVQZ basis set.

4.4 Problematic systems

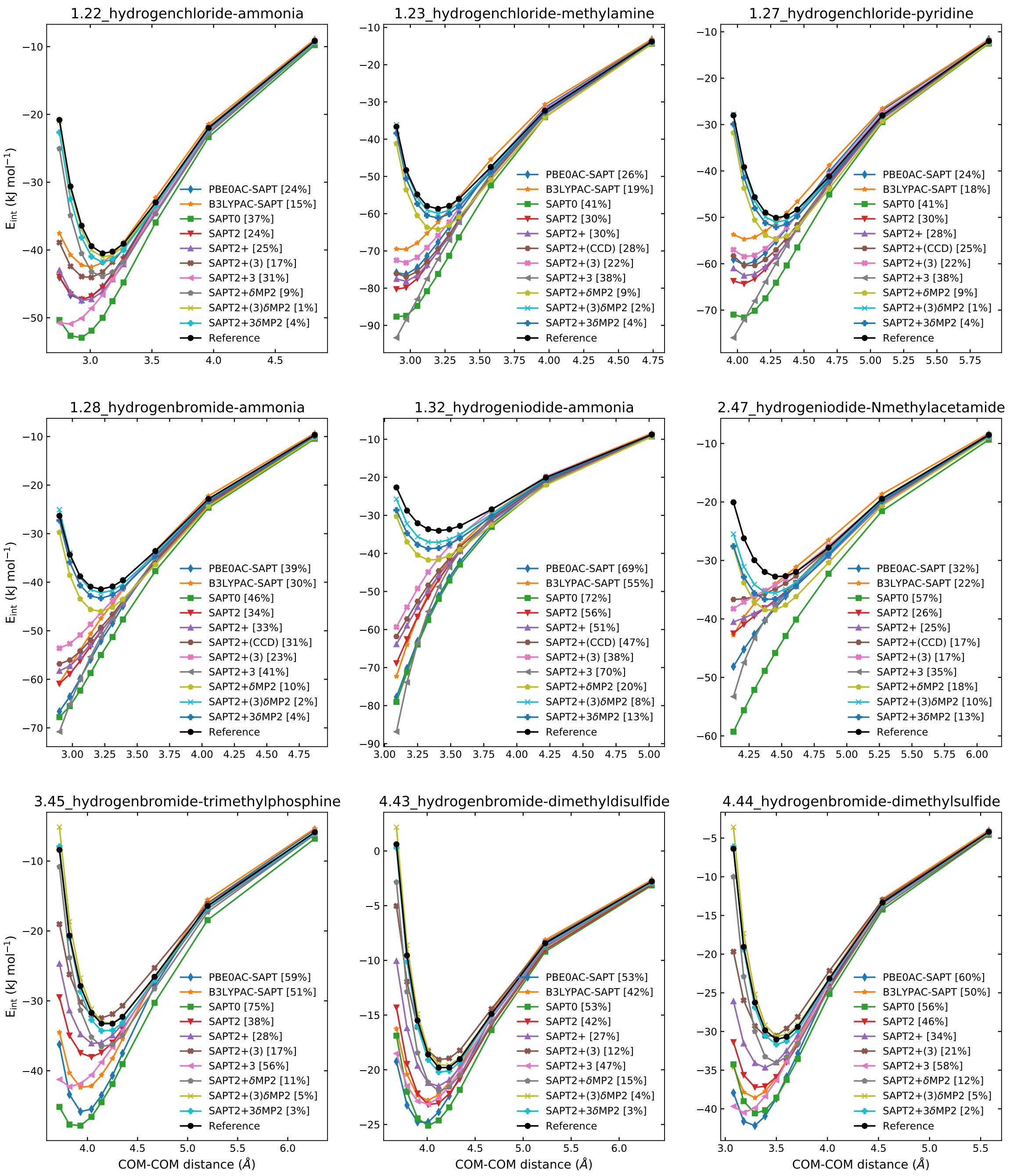

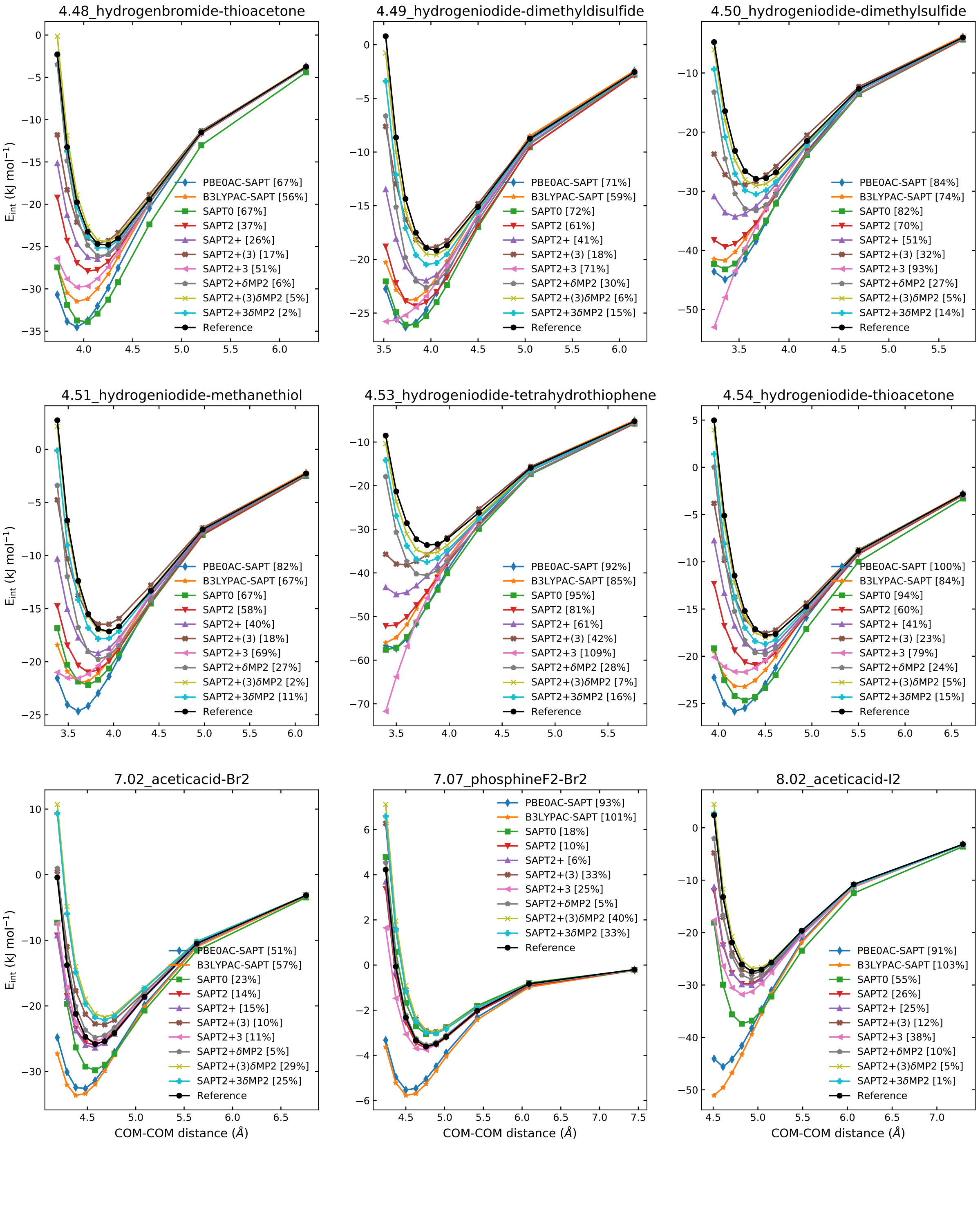
4.48_hydrogenbromide-thioacetone
PBE0AC-SAPT [67%]
B3LYPAC-SAPT [56%]
SAPT0 [67%]
SAPT2 [37%]
SAPT2+ [26%]
SAPT2+(3) [17%]
SAPT2+3 [51%]
SAPT2+δMP2 [6%]
SAPT2+(3)δMP2 [5%]
SAPT2+3δMP2 [2%]
Reference
4.49_hydrogeniodide-dimethyldisulfide
PBE0AC-SAPT [71%]
B3LYPAC-SAPT [59%]
SAPT0 [72%]
SAPT2 [61%]
SAPT2+ [41%]
SAPT2+(3) [18%]
SAPT2+3 [71%]
SAPT2+δMP2 [30%]
SAPT2+(3)δMP2 [6%]
SAPT2+3δMP2 [15%]
Reference
4.50_hydrogeniodide-dimethylsulfide
PBE0AC-SAPT [84%]
B3LYPAC-SAPT [74%]
SAPT0 [82%]
SAPT2 [70%]
SAPT2+ [51%]
SAPT2+(3) [32%]
SAPT2+3 [93%]
SAPT2+δMP2 [27%]
SAPT2+(3)δMP2 [5%]
SAPT2+3δMP2 [14%]
Reference
4.51_hydrogeniodide-methanethiol
PBE0AC-SAPT [82%]
B3LYPAC-SAPT [67%]
SAPT0 [67%]
SAPT2 [58%]
SAPT2+ [40%]
SAPT2+(3) [18%]
SAPT2+3 [69%]
SAPT2+δMP2 [27%]
SAPT2+(3)δMP2 [2%]
SAPT2+3δMP2 [11%]
Reference
4.53_hydrogeniodide-tetrahydrothiophene
PBE0AC-SAPT [92%]
B3LYPAC-SAPT [85%]
SAPT0 [95%]
SAPT2 [81%]
SAPT2+ [61%]
SAPT2+(3) [42%]
SAPT2+3 [109%]
SAPT2+δMP2 [28%]
SAPT2+(3)δMP2 [7%]
SAPT2+3δMP2 [16%]
Reference
4.54_hydrogeniodide-thioacetone
PBE0AC-SAPT [100%]
B3LYPAC-SAPT [84%]
SAPT0 [94%]
SAPT2 [60%]
SAPT2+ [41%]
SAPT2+(3) [23%]
SAPT2+3 [79%]
SAPT2+δMP2 [24%]
SAPT2+(3)δMP2 [5%]
SAPT2+3δMP2 [15%]
Reference
7.02_aceticacid-Br2
PBE0AC-SAPT [51%]
B3LYPAC-SAPT [57%]
SAPT0 [23%]
SAPT2 [14%]
SAPT2+ [15%]
SAPT2+(3) [10%]
SAPT2+3 [11%]
SAPT2+δMP2 [5%]
SAPT2+(3)δMP2 [29%]
SAPT2+3δMP2 [25%]
Reference
7.07_phosphineF2-Br2
PBE0AC-SAPT [93%]
B3LYPAC-SAPT [101%]
SAPT0 [18%]
SAPT2 [10%]
SAPT2+ [6%]
SAPT2+(3) [33%]
SAPT2+3 [25%]
SAPT2+δMP2 [5%]
SAPT2+(3)δMP2 [40%]
SAPT2+3δMP2 [33%]
Reference
8.02_aceticacid-I2
PBE0AC-SAPT [91%]
B3LYPAC-SAPT [103%]
SAPT0 [55%]
SAPT2 [26%]
SAPT2+ [25%]
SAPT2+(3) [12%]
SAPT2+3 [38%]
SAPT2+δMP2 [10%]
SAPT2+(3)δMP2 [5%]
SAPT2+3δMP2 [1%]
Reference
E_int (kJ mol⁻¹)
COM-COM distance (Å)

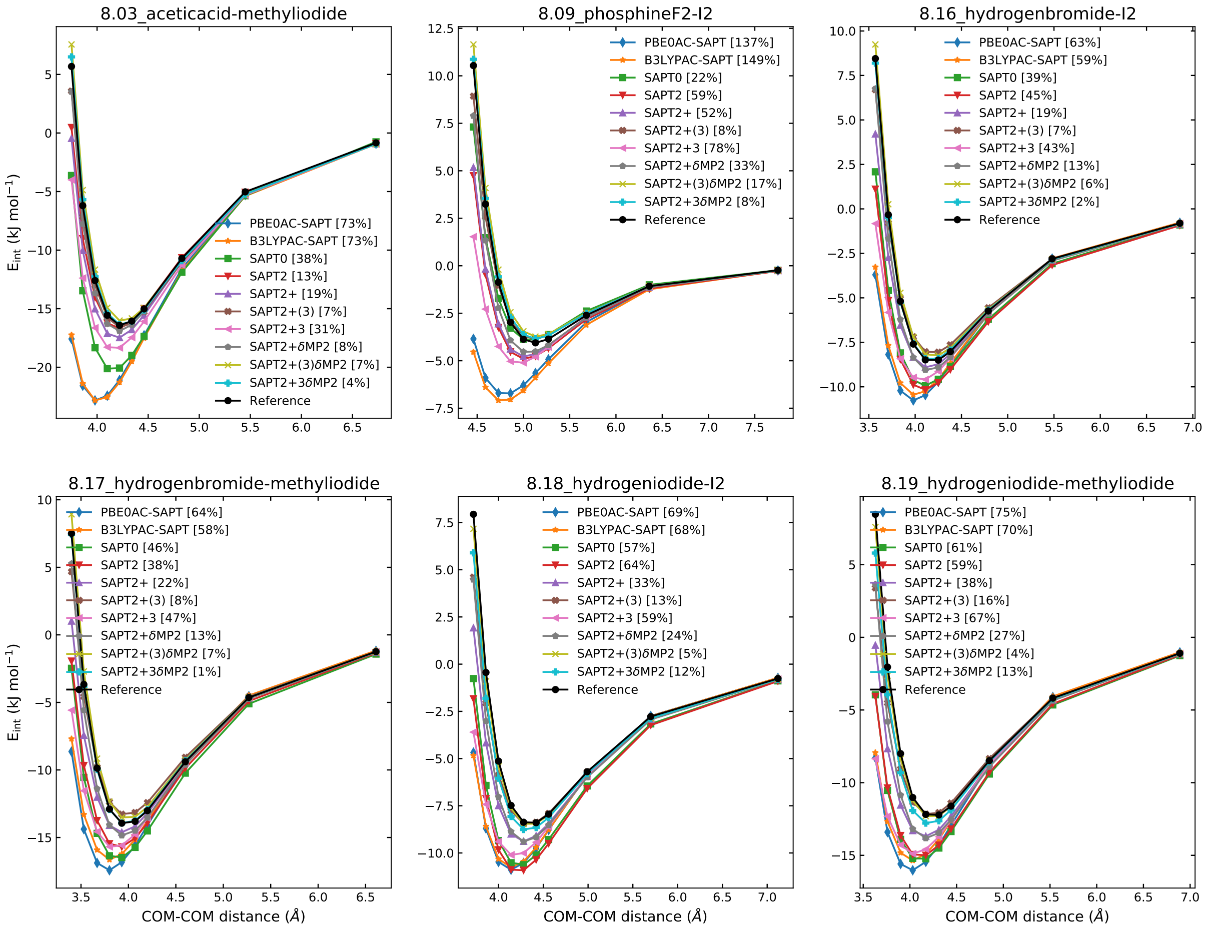

Figure S7: The comparison of the different variants of SAPT for the problematic systems in the HB300SPX×10 data set. The percentages in brackets are MCUREs for the whole dissociation curves related to each given method. The def2-QZVPPD basis set was used for all the SAPT methods in the Psi4 program packages. The DFT-SAPT results are at the complete basis set limit (CBS). The reference is CCSD(T)/CBS.

Table S6: Total MCUREs and RMSEs for different variants of SAPT applied on the problematic complexes.

Method	RMSE (kJ mol^{-1})	MCURE (%)
SAPT2+3δMP2/def2-QZVPPD	2.13	9.12
SAPT2+(3)δMP2/def2-QZVPPD	1.65	7.78
SAPT2+δMP2/def2-QZVPPD	3.43	16.36
SAPT2+3/def2-QZVPPD	14.08	53.45
SAPT2+(3)/def2-QZVPPD	6.89	18.92
SAPT2+/def2-QZVPPD	9.10	32.16
SAPT2/def2-QZVPPD	10.61	42.53
SAPT0/def2-QZVPPD	14.01	54.74
PBE0AC-SAPT/CBS	13.51	66.59
B3LYPAC-SAPT/CBS	11.99	61.07

4.5 Average of some energy terms

Table S7: the mean value of ind.disp^{-1}, ind, and charge transfer (CT) calculated with PBE0AC. The mean CT values are given only for two data sets, and the values in parentheses are related to longer range data points. Induction (ind) is equal to the sum of second order induction energy and its exchange component without δHF. Energies are in kJ mol^{-1}.

data set	subset[a]	ind.disp^{-1}	ind	ind%	CT	CT%
HB375×10		0.29	-6.77	40.75	-2.06(-0.31)	12.31(1.99)
	OH···N	0.55	-15.74	62.20	-6.21(-1.04)	25.61(4.79)
	OH···O	0.47	-12.34	54.05	-4.00(-0.63)	18.53(3.23)
	NH···N	0.30	-6.63	45.69	-2.13(-0.28)	16.20(2.38)
	NH···O	0.25	-5.27	37.51	-1.21(-0.16)	9.75(1.51)
	CH···N	0.27	-4.34	42.31	-1.12(-0.12)	12.91(1.58)
	CH···O	0.25	-3.94	37.79	-0.83(-0.10)	9.99(1.34)
	noHB	0.12	-2.08	24.90	-0.20(-0.01)	3.67(0.51)
D442×10		0.06	-1.64	25.53		
IHB100×10		1.99	-36.26	62.15		
SH250×10		0.24[b]	-10.41[b]	59.01[b]		
X40×10		0.183[b]	-3.63[b]	27.93[b]		
HB300SPX×10		0.42	-10.13	83.90	-4.68(-0.77)	39.23(3.43)
	AH···F	0.29	-4.91	50.34	-0.98(-0.08)	12.34(0.85)
	AH···Cl	0.28	-4.67	72.00	-1.54 (-0.18)	25.69(1.67)
	AH···Br	0.39	-7.73	108.63	-3.97(-0.54)	59.02(4.27)
	AH···I	0.49	-11.02	151.60	-6.23(-0.79)	86.1(6.02)
	AH···S	0.40	-11.35	91.38	-5.54(-0.85)	47.07(3.80)
	AH···P	0.35	-7.88	80.93	-3.59(-0.50)	36.15(2.72)
	AH···N	0.63	-17.24	87.57	-9.23(-1.90)	45.75(5.69)
	AH···O	0.55	-14.47	77.53	-6.61(-1.24)	36.33 (4.24)
R739×5		0.20	-2.79	66.97		
S66×8		0.22	-3.84	14.29		

[a]These subsets are extracted only from the related indicated data set

[b]These values were obtained through a regularized SAPT calculation

4.6 Intermolecular distances

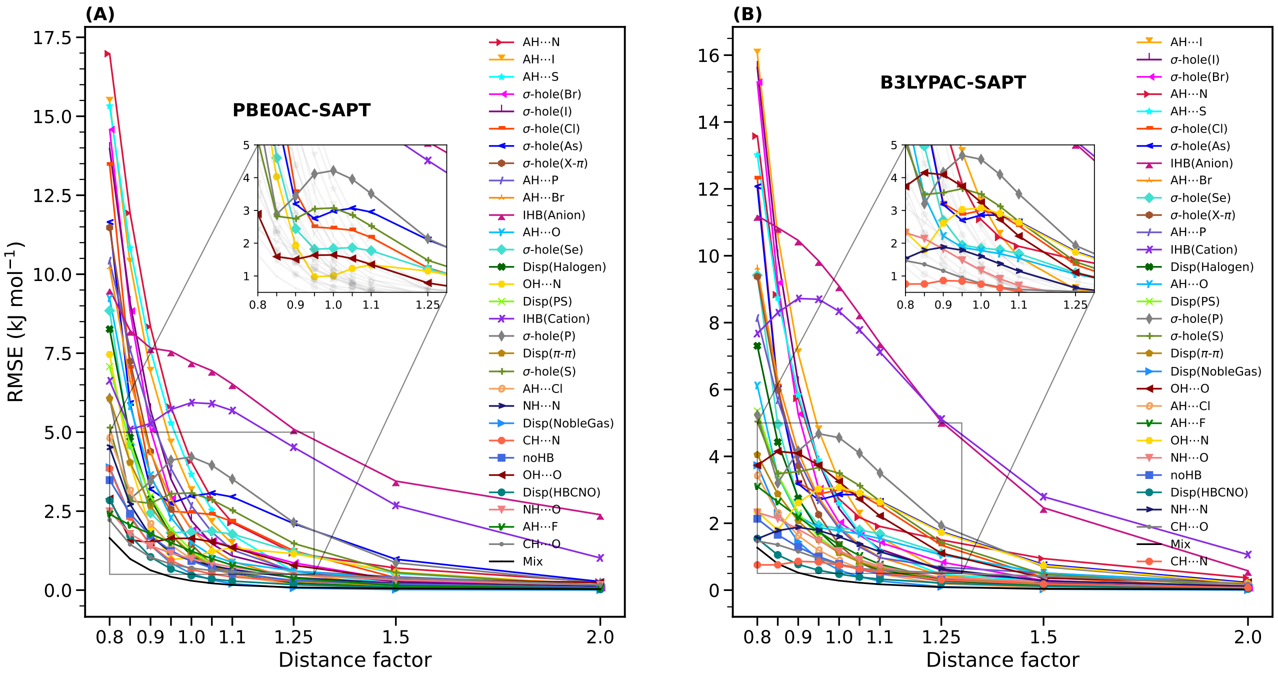

Figure S8: The RMSE value of SAPT calculations with PBE0AC and B3LYPAC (the inset plot highlights the non-smooth curves) for each group of interaction at every distance factor. The systems of R739×5 and S66×8 were excluded from these plots. The items in the legend were sorted based on the RMSE values at the smallest distance.

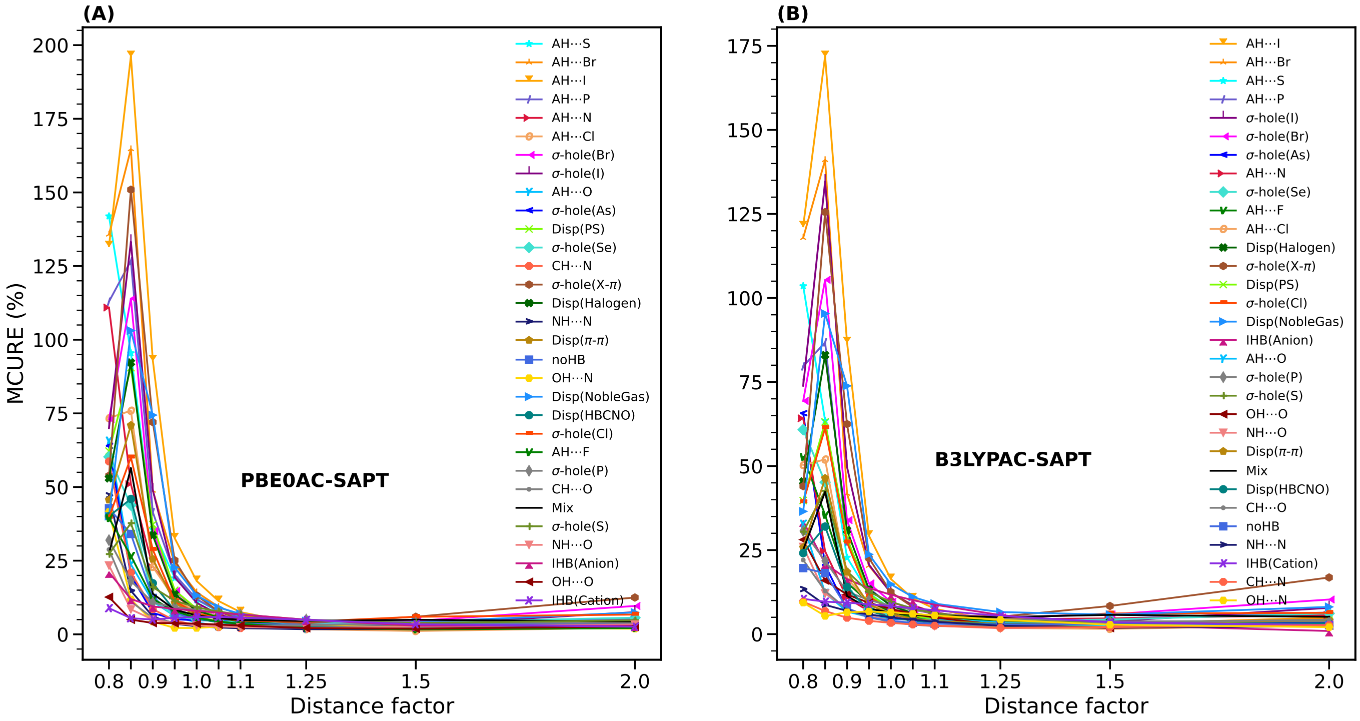

Figure S9: The MCURE percentages for each group of interaction at every distance factor. The systems of R739×5 and S66×8 were excluded from these plots. The items in the legend were sorted based on the RMSE values at the smallest distance.

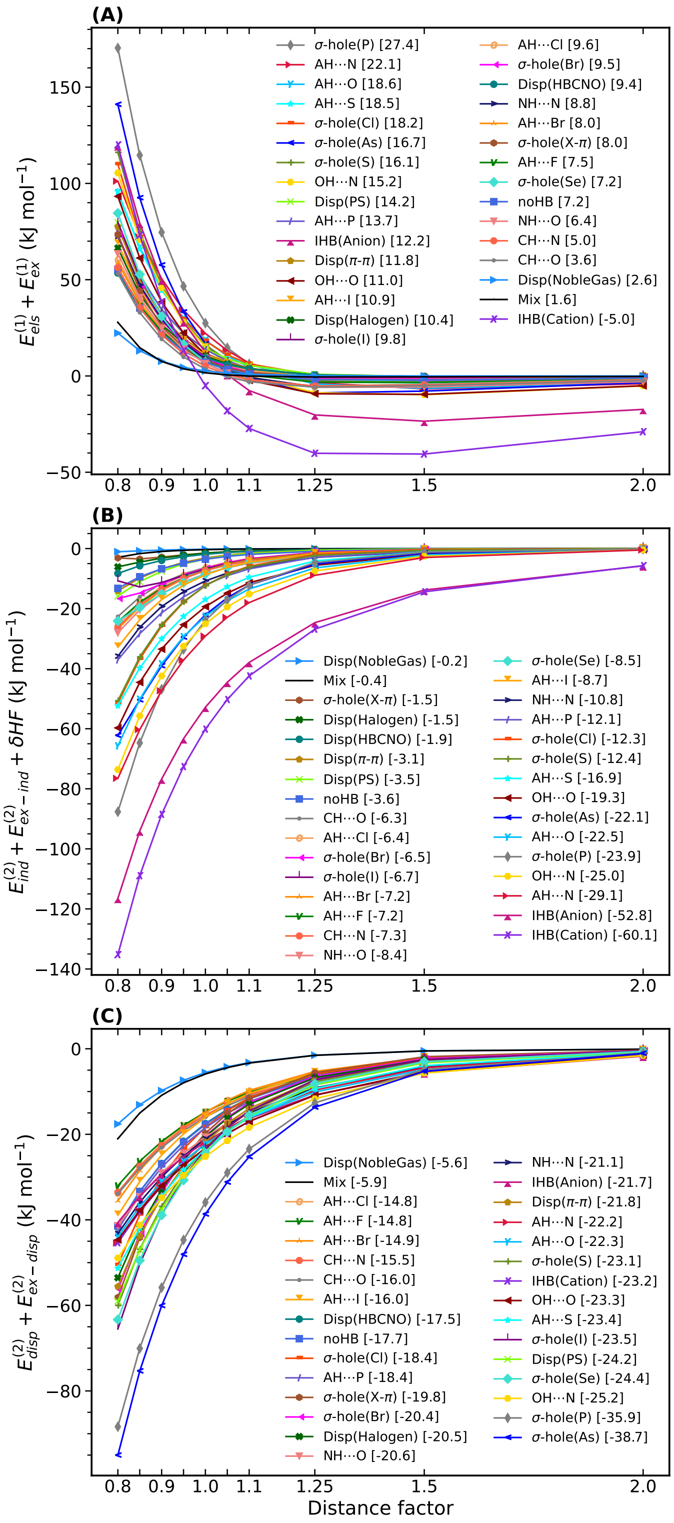

Figure S10: The average of energy components *vs.* the monomer separation. The values in brackets are the average of the corresponding term at the equilibrium point, and all the items in the legends are sorted based on these values. The systems of R739×5 and S66×8 were excluded from these plots. The underlying xc-potential is B3LYPAC here.

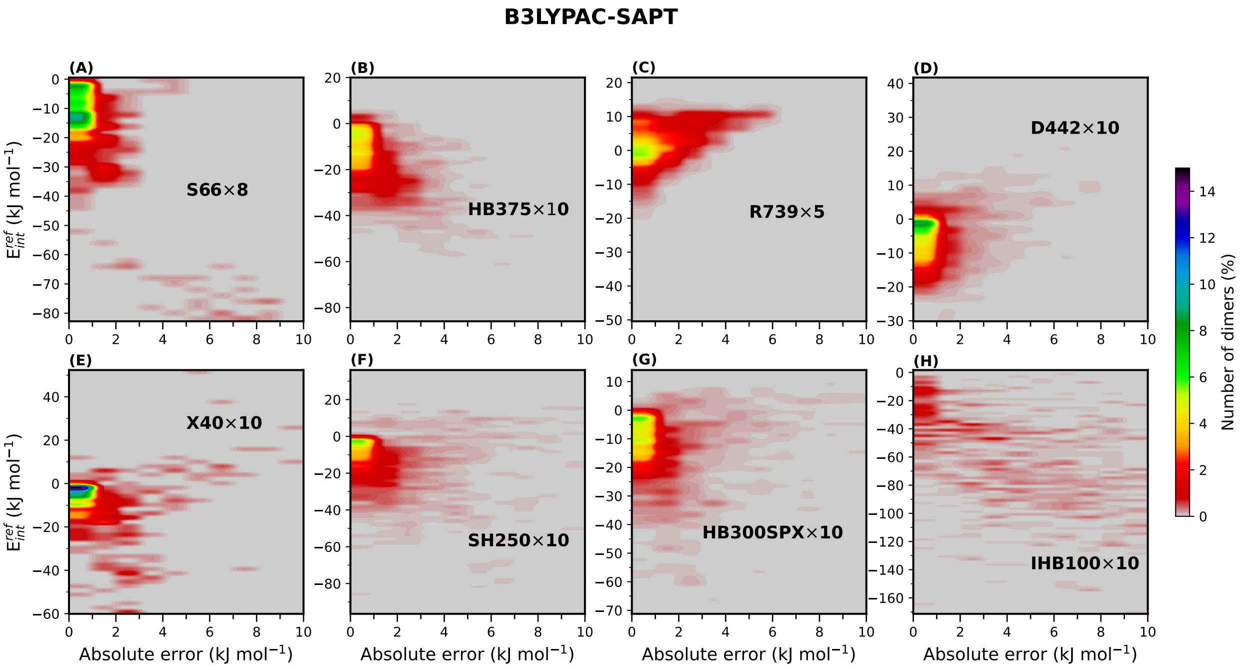

Figure S11: The percentage amount of individual dimers with a particular interaction energy (reference) and an unsigned error value for its corresponding B3LYPAC-SAPT energy for each data set.

4.7 Fit data

Table S8: The average of RMSE values in each category of interaction related to the fit of a single term function (C_nR^{-n})to dispersion data for both xc-potentials in kJ mol^{-1}.

Interaction group	PBE0AC-SAPT	B3LYPAC-SAPT
noHB+Mix	0.083	0.086
Disp(π-π)	0.101	0.106
Disp(Halogen)	0.093	0.097
Disp(PS)	0.103	0.109
Disp(NobleGas)	0.020	0.020
Disp(HBCNO)	0.069	0.073
CH$\cdots$N	0.054	0.057
NH$\cdots$O	0.077	0.081
AH$\cdots$F,Cl	0.061	0.065
IHB(Anion)	0.183	0.161
NH$\cdots$N	0.079	0.082
AH$\cdots$S,I	0.095	0.101
CH$\cdots$O	0.066	0.065
OH$\cdots$N	0.094	0.096
AH$\cdots$P,Br	0.076	0.081
Chalcogen bonds	0.086	0.092
OH$\cdots$O	0.079	0.080
AH$\cdots$N,O	0.093	0.099
Halogen bonds	0.072	0.097
Pnictogen bonds	0.135	0.141
IHB(Cation)	0.090	0.090

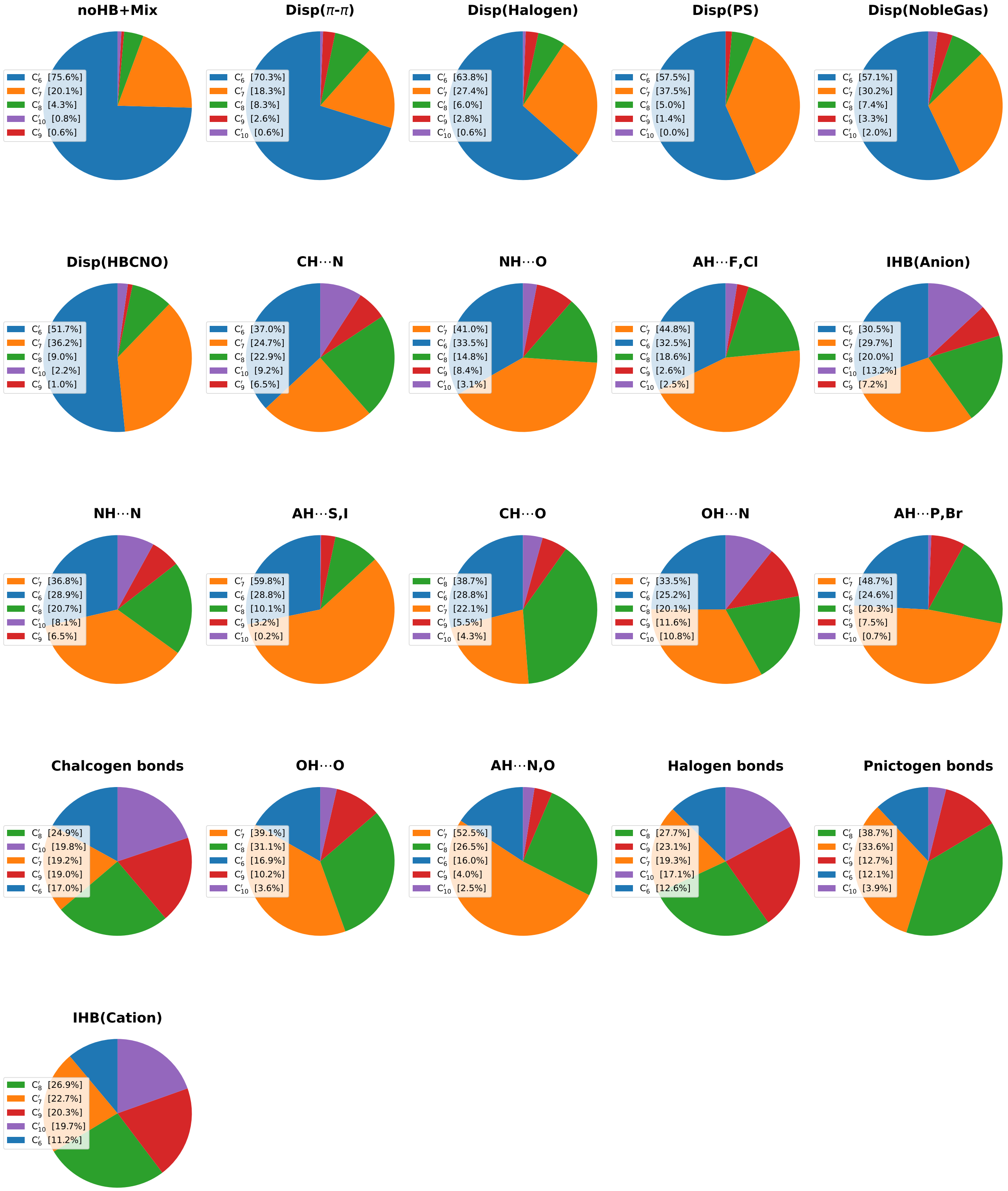

Figure S12: The mean contribution of each $\mathrm{C'_n}$ term in the expression $-\sum_{n=6}^{10} C'_n R^{-n}$ (fit to the dispersion energies obtained from PBE0AC-SAPT) to the whole dispersion energy for each interaction category.

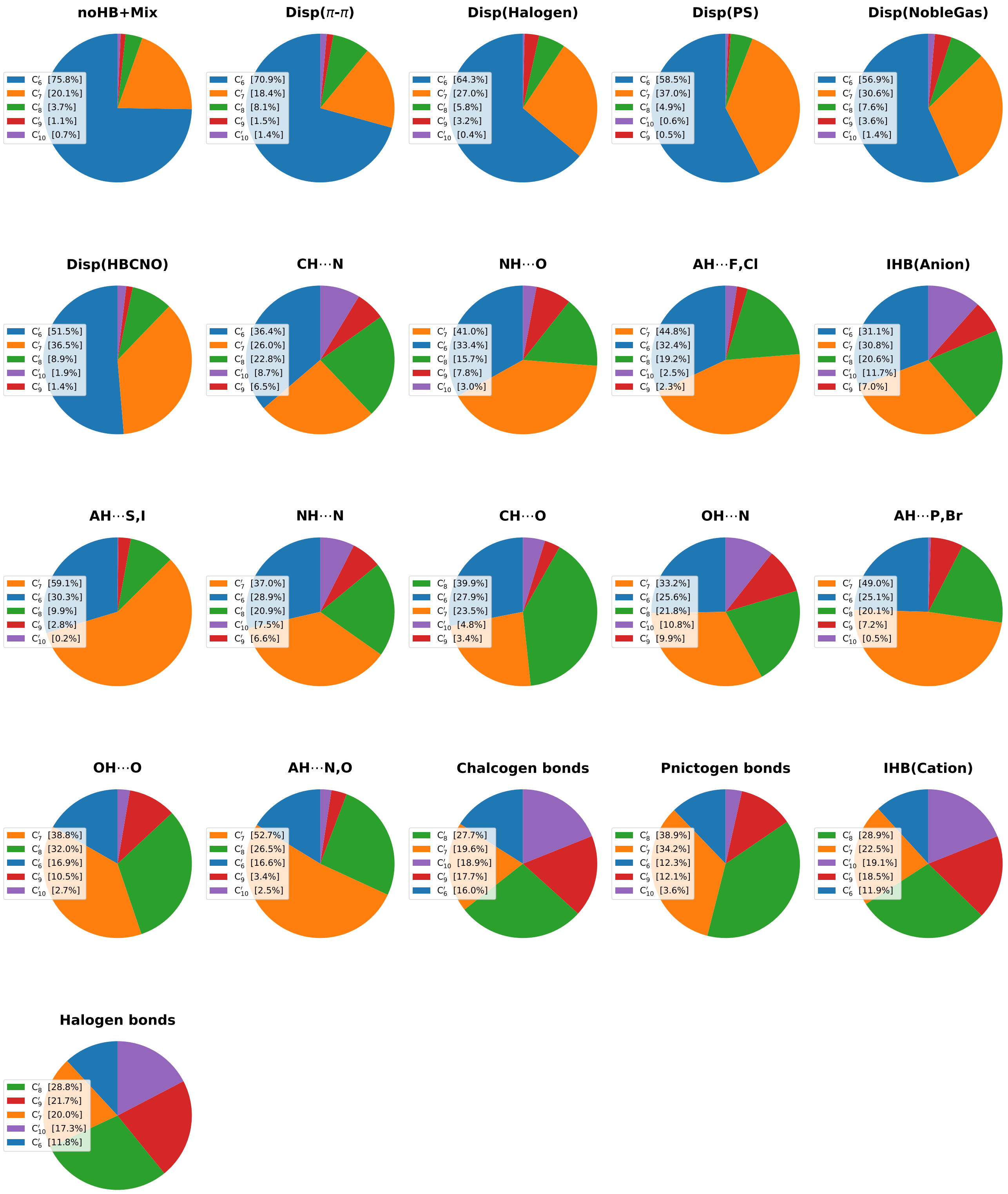

Figure S13: The mean contribution of each C'_n term in the expression $-\sum_{n=6}^{10} C'_n R^{-n}$ (fit to the dispersion energies obtained from B3LYPAC-SAPT) to the whole dispersion energy for each interaction category.

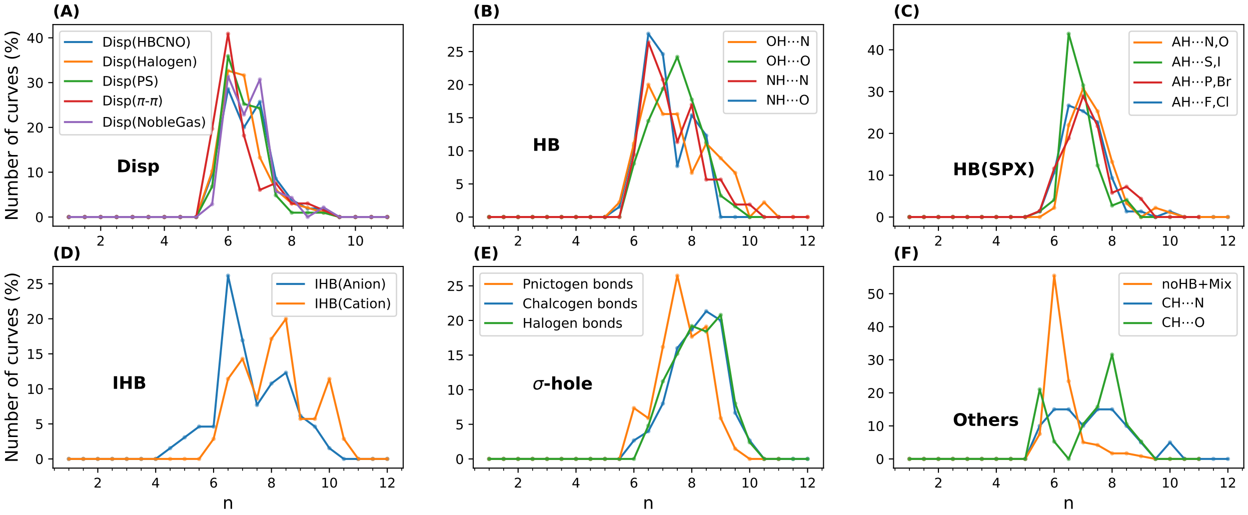

Figure S14: The number of dispersion energy curves (%) *vs.* the decay rate of dispersion indicated as n in R^{-n} for each category of interaction. The SAPT calculations were performed with the B3LYPAC potential.

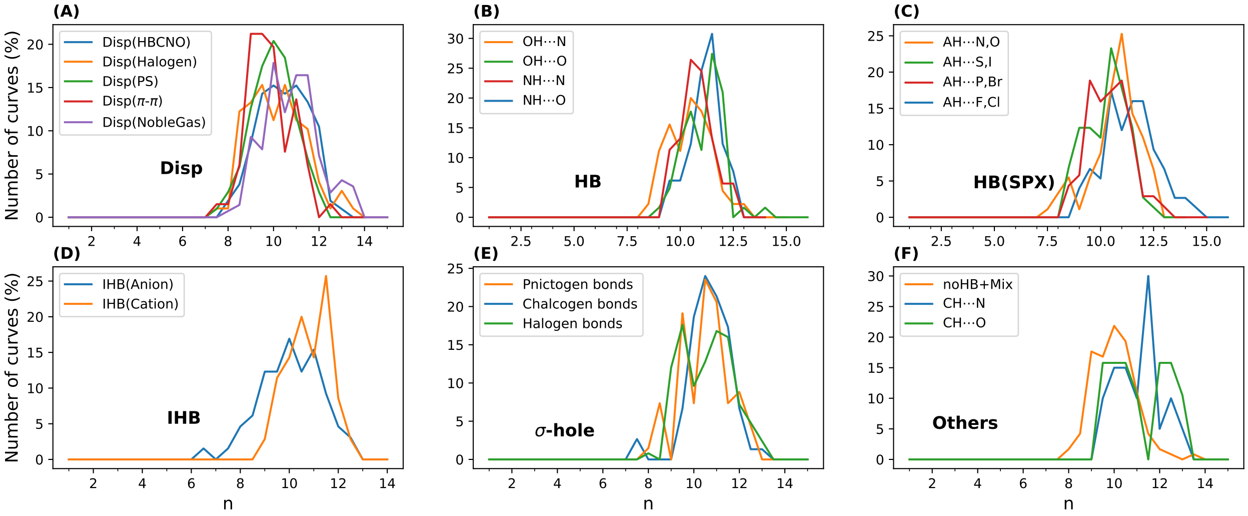

Figure S15: The number of exchange-repulsion curves (%) calculated with PBE0AC-SAPT described with the AR^{-n} model *vs.* n for each category of interaction.

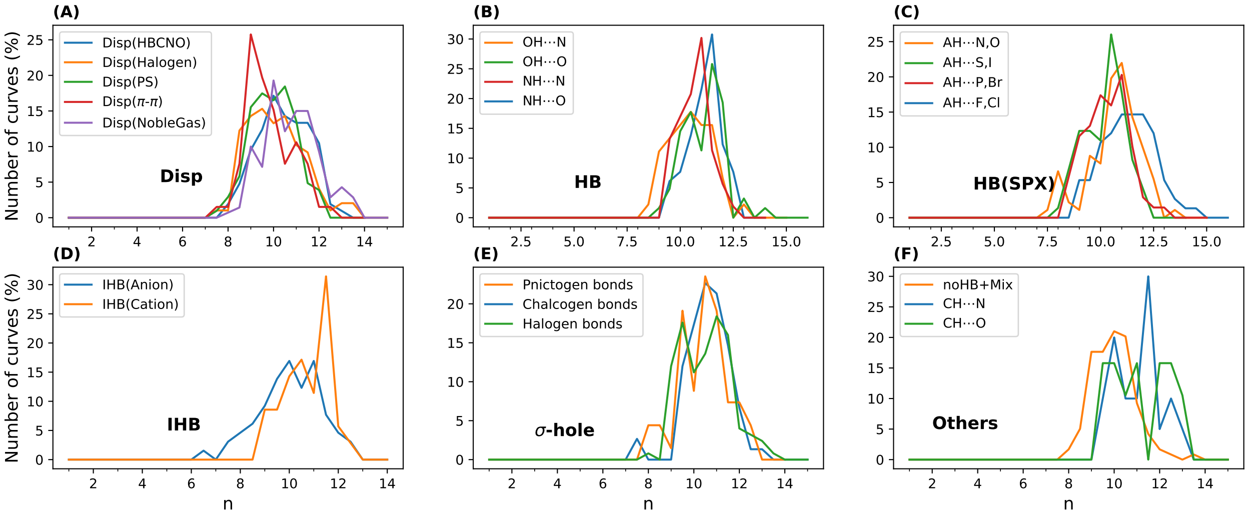

Figure S16: The number of exchange-repulsion curves (%) calculated with B3LYPAC-SAPT described with the AR^{-n} model *vs.* n for each category of interaction.

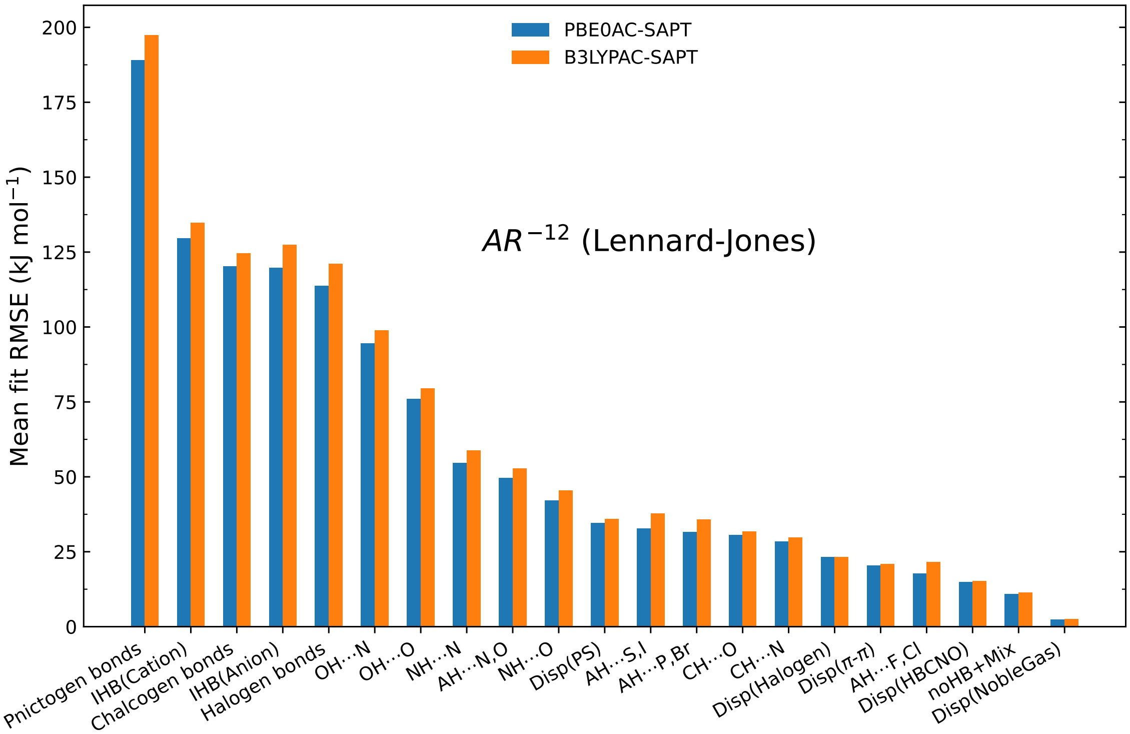

Figure S17: The average of RMSE values of the AR^{-12} fit to the exchange-repulsion values obtained from DFT-SAPT with two xc-potentials for different types of interaction.

4.8 Molpro *vs.* Psi4

Table S9: The comparison of the total RMSE (kJ mol^{-1}) and total MCURE percentages for the IHB100×10 data set computed with the Molpro and Psi4 programs. The PBE0AC results are of a relatively higher stability.

	**Psi4**		**Molpro**	
Method	RMSE	MCURE	RMSE	MCURE
PBE0AC	5.0	6.76%	5.16	5.85%
B3LYPAC	7.49	10.39%	4.23	5.11%

5 References

[1] Pan, F.; Wang, W.; Tung, A.; Yang, J. Finding representative set from massive data. *Fifth IEEE International Conference on Data Mining (ICDM'05).* 2005; pp 8 pp.–.

[2] Patkowski K. Chapter One - Benchmark Databases of Intermolecular Interaction Energies: Design, Construction, and Significance, in *Annual Reports in Computational Chemistry*, edited by D. A. Dixon (Elsevier, 2017) pp. 3–91.

[3] Smith D. G. A.; Burns L. A.; Patkowski, K.; Sherrill C. D. revised Damping Parameters for the D3 Dispersion Correction to Density Functional Theory. *J. Phys. Chem. Lett.* 2016, 7, 2197–2203.
